# COVID-19 Pandemic and Its Impact on Neurosurgery Practice in Malaysia: Academic Insights, Clinical Experience and Protocols from March till August 2020

**DOI:** 10.21315/mjms2020.27.5.14

**Published:** 2020-10-27

**Authors:** Azman Raffiq, Liew Boon Seng, Lim Swee San, Zaitun Zakaria, Ang Song Yee, Diana Noma Fitzrol, Wan Mohd Nazaruddin Wan Hassan, Zamzuri Idris, Abdul Rahman Izaini Ghani, Azmin Kass Rosman, Jafri Malin Abdullah

**Affiliations:** 1 Department of Neurosurgery, Penang General Hospital, Pulau Pinang, Malaysia; 2 Department of Neurosurgery, Hospital Sungai Buloh, Selangor, Malaysia; 3 Department of Neurosurgery, Sarawak General Hospital, Ministry of Health, Kuching, Malaysia; 4 Department of Neurosciences, School of Medical Sciences, Universiti Sains Malaysia, Kubang Kerian, Kelantan, Malaysia; 5 Department of Neurosciences, Hospital Universiti Sains Malaysia, Universiti Sains Malaysia, Kubang Kerian, Kelantan, Malaysia; 6 Department of Anaesthesiology and Intensive Care, School of Medical Sciences, Universiti Sains Malaysia, Kubang Kerian, Kelantan, Malaysia; 7 Brain and Behaviour Cluster, School of Medical Sciences, Universiti Sains Malaysia, Kubang Kerian, Kelantan, Malaysia; 8 National Head/Coordinator for Neurosurgical Services, Ministry of Health, Malaysia Care of Department of Neurosurgery, Hospital Sungai Buloh, Selangor, Malaysia

**Keywords:** coronavirus, neurosurgery, elective neurosurgery, emergency neurosurgery, tracheostomy, personal protective equipment, telecommunication

## Abstract

The newly discovered coronavirus disease 2019 (COVID-19) is an infectious disease introduced to humans for the first time. Following the pandemic of COVID-19, there is a major shift of practices among surgical departments in response to an unprecedented surge in reducing the transmission of disease. With pooling and outsourcing of more health care workers to emergency rooms, public health care services and medical services, further in-hospital resources are prioritised to those in need. It is imperative to balance the requirements of caring for COVID-19 patients with imminent risk of delay to others who need care. As Malaysia now approaches the recovery phase following the pandemic, the crisis impacted significantly on neurosurgical services throughout the country. Various emergency measures taken at the height of the crisis may remain as the new normal in the provision of neurosurgical services and practices in Malaysia. The crisis has certainly put a strain on the effective delivery of services and as we approach the recovery era, what may have been a strain may prove to be a silver lining in neurosurgical services in Malaysia. The following details are various measures put in place as the new operational protocols for neurosurgical services in Malaysia.

## Introduction

The novel coronavirus, otherwise known as coronavirus disease 2019 (COVID-19) pandemic in Malaysia, is part of the worldwide ongoing pandemic which was first reported in Wuhan China in December 2019. Malaysia’s first case was confirmed on 25 January 2020, detected among travellers from China entering Malaysia via Singapore. Malaysia’s response overseen by the National Crisis Preparedness and Response Centre (CPRC) under the Health Ministry began as early as 6 January 2020 following World Health Organization (WHO) initial report of the disease in China. Various hospital was designated specifically as COVID-19 pandemic hospitals, gazettement of quarantine centres, reorganisation of health services, formulation of new operational protocols, stockpiling of essential equipment and redeployment of essential medical workforce and manpower to these centres; as well active detection, monitoring and treating COVID-19 patients. These early response measures were escalated to a total lockdown of movement on 18 March 2020, intended to mitigate the spread of COVID-19, following the declaration of a worldwide COVID-19 pandemic by WHO on 11 March 2020. The Movement Control Order (MCO) in Malaysia lasting for a period of 6 months, coupled by the intense proactive measures by the Health Ministry has successfully kept the number of cases relatively low comparatively, with a total recorded case of 8,556 with 121 deaths. Malaysia’s proactive and stringent measures have and continue to receive international praise and recognition in successfully mitigating the spread and containing COVID-19 transmission.

As Malaysia now approaches the recovery phase following the pandemic, the crisis impacted significantly on neurosurgical services throughout the country. Various emergency measures taken at the height of the crisis may remain as the new normal in the provision of neurosurgical services and practices in Malaysia. The crisis has certainly put a strain on the effective delivery of services, and as we approach the recovery era, what may have been a strain may prove to be a silver lining in neurosurgical services in Malaysia. The following details the various measures put in place as the new operational protocols for neurosurgical services in Malaysia.

## Role and Importance of Dedicated Operating Theatre, Instruments and ICU Care

### Dedicated Operating Theatre

All tertiary hospital should have a dedicated operating theatre (OT) for suspected COVID-19 patients (1). The OT requirements should be fully equipped with negative pressure ventilation (recommended). The maximal number of staff is six: i) one specialist; ii) one medical officer; iii) one anaesthetist; iv) one anaesthetist MO; v) one scrub nurse; and vi) one circulating nurse. For elective surgery, kindly refer to its section further below (2). Limiting OT staff to essential members will help preserve the surgical workforce. The types of neurosurgery cases done are either cranial surgery or spine surgery. Endonasal surgery should be avoided during COVID-19 pandemic (3). Table 1 shows tiers for neurosurgery cases (4). A neurosurgical emergency includes cerebral haemorrhages (subarachnoid and intraparenchymal), acute hydrocephalus, tumours at risk of intracranial hypertension, spinal cord compressions with, or at risk of neurological deficit, and traumatic cranial and spinal trauma emergencies (5).

### Distribution of neurosurgery operative cases (Table 2) (6)

For green level, all elective cases proceed as scheduled. For yellow level, the OT schedule is capped for 3 weeks to 75% of capacity, yielding a 25% reduction in all elective and procedural cases. All outpatient procedures should be designated to an off-site hospital where COVID-19 patients are not expected to be admitted. There is a hard cap on the number of cases requiring post-operative admission; the 12-patient limit for all surgical cases (including non-neurosurgical cases). For red level, there is a 50% reduction in all elective case scheduling. For black level, significant state or federal resources are needed to fight the outbreak. All urgent scheduled surgical cases will be cancelled. This ‘volume limiting approach’ encourages maximal adaptability, in which the supply of hospital capability meets the demand for scheduling needs.

### A 3-group setting (surgical team) (Table 3) (6)

The team-based paired coverage will go into effect during red levels of COVID-19. In this model, each hospital (columns) will have three groups of providers. There will be two teams that switch coverage on a 3-day cycle. Each team will cover for 3 days, and then have 3 days off while the second team covers. The transition between teams will occur virtually, avoiding unnecessary team-to-team contact. A backup group substitutes for any team member who shows signs of illness. If a team becomes contaminated, the other team will take over and the alternates will fill the gap. Contact between teams and alternate is prohibited. Each team will only rotate at one hospital (no cross-covering) and will only have contact with members within their team. Teams at the same site will not have any overlapping clinical time with each other. This system ensures adequate coverage, minimises hand-off issues, and, most importantly, minimises transmission risk across teams. Due to the likelihood of infection among inpatient providers, there will be a designated ‘alternate pool’ of providers that will substitute for those who show COVID-19 symptoms.

**Figure 1:**
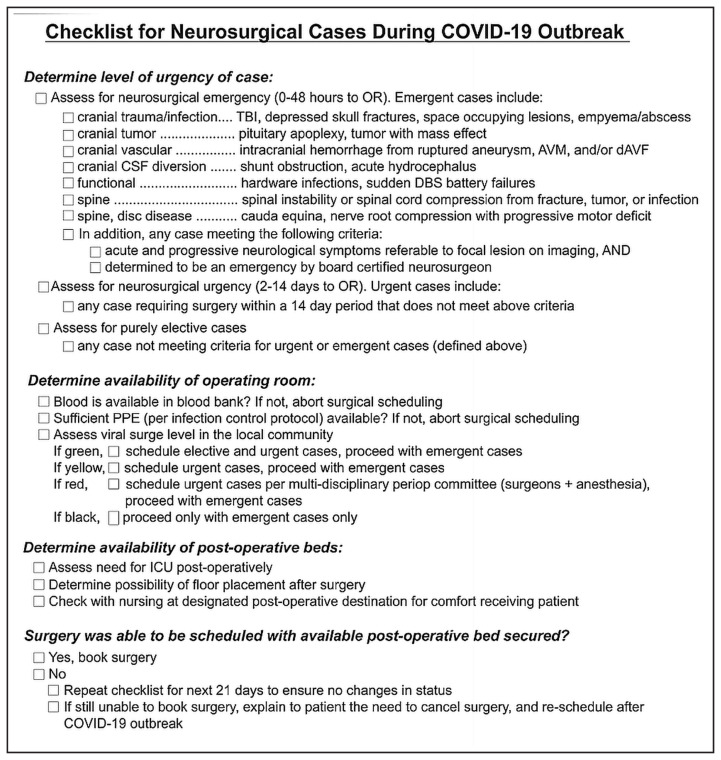
Checklist for neurosurgical cases during COVID-19 outbreak

Activation of paired coverage system (PCM) (6) is triggered by a red level of surge. All residents are aware of their role in the PCM ahead of time. Site-specific needs are addressed within the team. For example, teams at the main hospital are larger than teams at other satellite hospitals. The PCM is adaptable such that the number of team members can vary, along with the experience level of the team. The core function of the PCM (limiting healthcare worker transmission of the virus) remains. It is also important that neurosurgeons provide their anaesthesia colleagues, nursing staff and the OT with objective data about which cases should be expected to proceed during a COVID-19 outbreak. Neurosurgeons need to predict what cases should be classified as an emergency. A checklist that can be applied to neurosurgical cases during the COVID-19 pandemic is as per Figure 1. The checklists help organise surgical staff during times of crisis, such as guiding action during ‘red alerts’ from neuromonitoring during spinal surgery. The checklist strategy organises surgical staff around the common goal of booking cases during the outbreak. Distribution of the checklist to all surgical staff will facilitate effective communication and the ease with which appropriate neurosurgical cases can be scheduled.

### Surgical workforce (7)

The surgical workforce must be able to maintain emergency surgery capabilities including major trauma. It is important to protect and preserve the surgical workforce as well. These will include rotas where some members of the team do not come into work and act as a healthy reserve (refer to a 3-group setting [surgical team]). When not vital to the effort, keep surgical and anaesthetic staff out of hospital and self-isolating at home to preserve human resources. This will also allow personnel to rest before they return to clinical work. Non-surgical solutions to be used to avoid surgery where possible. Personal protective equipment (PPE) must be used correctly in line with national guidance. Rest, recuperation and psychological support should be factored into all planning.

### Screening and requirement for urgent/emergent cases (3, 8, 9)

Pre-operatively, the patient must be tested for COVID-19 and proceed if negative. Full powered air purifying respirators (PAPRs) for surgeons and all team members in the OT for any of these cases that do actually need to move forward, either for cases in which we cannot wait for test results or for cases that test positive but still need to proceed.

For urgent cases (that should be done within 1 week), two COVID-19 tests separated by 24 h with the patient quarantined in the interval between tests before the surgery, with the surgery proceeding only if the results are negative for both tests. If COVID positive, PAPR for all OT staff may be necessary until further data is available.

For emergent/unavoidable case for a known or undetermined COVID-19 patient, the surgeon and all OT personnel in the surgical suite should use PAPR, which filter the air being breathed in addition to face shields and other standard PPE. It is also vital for a cessation of positive pressure ventilation in OT during the procedure until 20 min after the patient leaves (10). The patient should be isolated and medical personnel should wear full PPE when nursing post-operative patients until COVID-19 status is known.

### Workup before surgery in pandemic COVID-19 (11)

Patient should be screened for FTOCC (fever, travel, occupation, contact and clustering) histories, upper respiratory and gastrointestinal tracts symptoms, anosmia, body temperature, chest X-ray, nasopharyngeal swab or throat swab. Patient should be kept while waiting for result before surgery. Patient should wear mask and strict hygiene. Blood test is done to look for leukopenia, lymphopenia, raised creatine kinase, lactate dehydrogenase and C-reactive protein. If none of above, then categorise as ‘standard risk’ otherwise ‘high risk’. High risk requires full PPE. (Refer to the additional document: Recommended PPE to be Used When Managing PUI or Confirmed COVID-19 — KKM 26 March 2020).

### Anaesthesia (1, 3)

Regional anaesthesia (RA) is preferred than general anaesthesia (GA) to reduce aerosols. In GA, a negative pressure setting (risk of aerosol transmission) is required. The intubation and extubation are done with full PPE including PAPR or its equivalent. Only essential staff should be present in the OT. Post-op management is done in the isolation room. Thromboprophylaxis should be considered throughout the hospital stay. Non-invasive ventilation should not be used.

### Anaesthesia work environment (1)

Proper droplet precautions or proper decontamination processes should be followed. There should be an escalation of standard of practice during airway management for all patients to reduce exposure to secretions. Hand hygiene should be taken care of with frequent hand washing using alcohol-based hand wash gels, which should be available near every anaesthesia station. The number of staff members present for intubations/extubations should be limited to reduce the risk of unnecessary exposure. It is also recommended to strongly consider prophylactic antiemetics to reduce the risk of vomiting and possible viral spread.

### Airway management (3, 9, 12)

Rapid sequence induction (RSI) and use a video laryngoscope (VL) with the goal of a high first pass success rate (FPS) is recommended. IV sequence induction without bag mask ventilation is preferred to minimise exposure risks and aerosolisation. Preoxygenation for a minimum of 5 min with 100% oxygen and perform a RSI to avoid manual ventilation of patient’s lungs and potential aerosolisation of virus from airways (3). RSI (ensure a skilled assistant is available to perform cricoid pressure) or a modified RSI should be performed as clinically indicated. If manual ventilation is required, apply small tidal volumes (3). Placement of a high quality heat and moisture exchanging filter (HMEF) rated to remove at least 99.97% of airborne particles 0.3 microns or greater in between the facemask and breathing circuit or in between facemask and reservoir bag is required. Awake fibreoptic intubations are essentially contraindicated unless specifically indicated.

PPE should be provided when performing an aerosol generating procedure. Preoxygenation using a bag-valve-mask (BVM) that can be purposely modified for COVID-19 patients with a viral filter, without squeezing the bag. The most experienced anaesthesia professional available should perform intubation if possible. A trainee should avoid intubations of sick patients during this time. The laryngoscope should be re-sheathed immediately post-intubation (double glove technique). Used airway equipment should be sealed in a double zip-locked plastic bag and must then be removed for decontamination and disinfection. There should be no airway carts in the room. Thus, appropriately sized equipment should be pre-packed for that patient. The use of deeper sedation extubation to prevent coughing is also preferable as long as the airway is safe.

### PPE by anaesthesia (3, 12, 13)

All anaesthesia professionals should utilise PPE appropriate for aerosol-generating procedures for all patients when working near the airway. ‘Rescue like’ crash intubations where PPE cannot be fully adhered to should be avoided. Correct donning and doffing of PPE should be adhered to. Properly fitted N95 masks or PAPRs should be used for all patient. At a minimum, N95 masks should be used for all patients. For those who are not N95 fit-tested, have facial hair or fail N95 fit-testing, PAPRs should be used if possible. Issuance of N95 masks or availability of PAPRs for all clinical anaesthesia personnel should be a priority. Extended use and/or limited reuse of N95 masks should follow the Centres for Disease Control and Prevention (CDC) and Institutional guidelines. A PAPR provides superior protection and may be warranted for airway procedures in patients with known or suspected COVID-19.

For aerosol-generating procedures, this includes eye protection (goggles or a disposable face shield that covers the front and sides of the face), a gown and gloves, in addition to airway protection with N95 masks or PAPRs. Effective hand hygiene before putting on and after removing PPE must be ensured. Procedures for proper donning and doffing, disposal of contaminated PPE, and cleaning of contaminated reusable PPE and anaesthesia equipment should be established following CDC and institutional recommendations. The double gloving technique is used during intubation. The outer gloves are used to sheath the laryngoscope blade and change the inner gloves as soon as possible afterwards. After removing protective equipment, remember to avoid touching your hair or face before washing hands. During extubation, maintain strict hands hygiene, wear a mask with a face shield and carefully dispose of contaminated equipment. The use of two providers for PPE donning and doffing procedures should be encouraged, to allow one person to observe and coach the other through the steps of the routine. Appropriate PPE and the procedures for donning and doffing PPE are available at the CDC webpage.

### Approaches to conserve supplies (12, 13)

The administration should minimise the number of individuals involved. Where feasible, use alternatives to N95 masks (e.g. other classes of filtering facepiece masks, facepiece air purifying respirators and PAPRs). N95 masks should be allowed for extended use and/or limited reuse. The use of N95 masks should be prioritised for that personnel at highest risk of COVID-19 exposure and/or those anaesthesia professionals in high risk categories (e.g. those with prior health conditions, older age). Staff should receive training in the appropriate donning and doffing of PPE taught through simulation and videos without using precious resources. In resource-limited situations, extended use of N95 masks (continuous wearing while seeing multiple patients) is preferred to limit the reuse of N95 masks (doffing and redonning between patients).

N95 mask life may be lengthened and surface contamination reduced by wearing a plastic face shield or a surgical mask over the N95 (CDC respirator guidance). Use of chlorine or alcohol solution to sanitise N95 masks is not recommended as it damages mask integrity. Heating N95 masks to 70 °C (160 °F) in a dry oven for 30 min seems a promising solution to disrupt viral particles and maintain mask integrity for reuse. Further guidance from CDC and Partners in Health (PIH) on reuse of N95 masks or best practices when no respirators are available (such as wearing two surgical masks) are available.

In routine clinical care of COVID-19 suspected or confirmed infections, surgical masks are acceptable PPE, except in the case of aerosol generating procedures (intubation, high flow nasal cannula, non-invasive ventilation, bronchoscopy, administration of nebulised medications, etc). Facilities which do not have disposable surgical attire, theatre garb in the form of cloth scrub hats or bonnets should be washed between each use if possible and no less than daily. Theatre gowns and drapes should be washed and sterilised between each patient as is currently expected. If theatre gowns are repurposed for isolation units, they should be washed after each prolonged care routine. If surgical PPE is not impermeable, consider wearing rubber aprons under linen gowns and always perform handwashing after doffing surgical PPE and before touching clean items or self.

### OT management and preparation during COVID-19 (14, 15)

Surveillance on possible further transmission to patients and other personnel should be done. COVID-19 in a patient receiving surgery is sporadically reported with a special focus of management technique. Surgical infection usually focuses on patient but it is important to give attention to the practitioner who works in the operation room.

An OT with a negative room pressure environment located at a corner of the operating complex, and with separate access, is designated for all confirmed (or suspected) COVID-19 cases. The OT consists of five interconnected rooms, of which only the anteroom and anaesthesia induction rooms have negative atmospheric pressures. The OT proper, preparation and scrub rooms all have positive pressures. Understanding the airflow within the OT is crucial to minimising the risk of infection. The same OT and the same anaesthesia machine will only be used for COVID-19 cases for the duration of the epidemic. An additional heat and moisture exchanger (HME) filter are placed on the expiratory limb of the circuit. Both HME filters and the soda lime are changed after each case.

The anaesthetic drug trolley is kept in the induction room. No unnecessary items should be brought into the OT, including personal items such as mobile phones and pens. Before the start of each operation, the anaesthesiologist puts all the drugs and equipment required for the procedure onto a tray to avoid handling of the drug trolley during the case. Nevertheless, if there is a need for additional drugs, hand hygiene and glove changing are performed before entering the induction room and handling the drug trolley. A fully stocked airway trolley is also placed in the induction room, as far as possible, disposable airway equipment is used. If single-use plastic anaesthesia or surgical equipment (endotracheal tubes, ventilator circuit tubing, plastic suction tubing, electrocautery handpieces) must be reused, ensure that disinfection aiming for ‘high-level disinfection’ or ‘sterility’ is employed. This includes immersion in appropriate concentration glutaraldehyde, phenol, or hydrogen peroxide solution for the recommended duration.

The airway should be secured using the method with the highest chance of first-time success to avoid repeated instrumentation of the airway, including using a video-laryngoscope. Equipment in limited supply such as bispectral index monitors or infusion pumps may be requested but need to be thoroughly wiped down after use. Hospital security is responsible for clearing the route from the ward or intensive care unit (ICU) to the OT, including the elevators. Traffic should be minimised, especially opening and closing of theatre doors. Patients with known or suspected COVID-19 infection should wear surgical masks when being transported through hospital spaces or in rooms without negative pressure isolation. The transfer from the ward to the OT will be done by the ward nurses in full PPE including a well-fitting N95 mask, goggles or face shield, splash-resistant gown and boot covers. For patients coming from the ICU, a dedicated transport ventilator is used to avoid aerosolisation, the gas flow is turned off and the endotracheal tube clamped with forceps during the switching of ventilators. The ICU personnel should wear full PPE with a PAPR for the transfer.

In the induction room, a PAPR is worn during induction and reversal of anaesthesia for all personnel within 2 m of the patient. For operative airway procedures such as tracheostomy, all staff keep their PAPR on throughout the procedure. During the procedure, a runner is stationed outside the OT if additional drugs or equipment are needed. These are placed onto a trolley that will be left in the anteroom for the OT team to retrieve. This same process in reverse is used to send out specimens such as arterial blood gas samples and frozen section specimens. The runner wears PPE when entering the anteroom. Personnel exiting the OT discard their used gowns and gloves in the anteroom and perform hand hygiene before leaving the anteroom. Any PAPR will be removed outside the anteroom. Patients who do not require ICU care post-operatively are fully recovered in the OT itself.

When the patient is ready for discharge, the route to the isolation ward or ICU is again cleared by security (using an advance runner to clear the way). A minimum of 1 h is planned between cases to allow OT staff to send the patient back to the ward, conduct thorough decontamination of all surfaces, screens, keyboard, cables, monitors and anaesthesia machine. Surfaces in the OT should be thoroughly cleaned between cases including pulse oximeter probes, thermometers, blood pressure cuffs and other reusable materials (70% alcohol solution or 0.5% chlorine solution).

As part of minimising contamination in OT, in addition to surface cleaning, using clear plastic sheets (to be changed in between patients) to cover the anaesthesia machine, the monitors as well as the patient’s face, especially during aerosol producing airway manoeuvres like intubation and extubation, is recommended. All unused items on the drug tray and airway trolley should be assumed to be contaminated and discarded. All staff must shower before resuming their regular duties. As an added precaution, after confirmed COVID-19 cases, a hydrogen peroxide vaporiser will be used to decontaminate the OT. Clear instructional posters for PPE donning/doffing should be prominently displayed. A taped off area just outside of the OT door should be clearly marked for donning and doffing activities.

### Minimising risks among health care providers (HCPs) (16)

Few steps must be emphasised to reduce the risk of contracting COVID-19 among HCPs. Some precaution that needs to be taken note are:

- Removing all jewellery and watches during patient care
- Not wearing nail polish
- Performing frequent hand hygiene with an alcohol-based hand wash or with soap and water for at least 20 s
- Avoiding touching your face
- Sanitising your workstation, stethoscope and phone throughout your shift
- Practicing respiratory hygiene
- Maintaining physical distance (a minimum of 2 m) from persons with respiratory symptoms or anyone who is at risk of being infected
- Changing into clean clothing before leaving work and showering in the hospital or immediately upon arrival to your home
- Those exhibiting infectious symptoms should immediately self-isolate and contact their administrators to discuss COVID-19 testing
- Targeted administrative, environmental and engineering controls should be implemented to reduce the risk of exposure
- Ensure appropriate infrastructure, testing capacity, zoning of patients according to risk of disease, and adequate staffing levels and training
- Engineering interventions such as physical separation of staff from patients where possible (e.g. the use of telephone or intercom communications) and appropriate ventilation (e.g. negative pressure rooms for aerosolising procedures)
- Walled rooms for aerosol-generating medical procedures (AGMP) with appropriately sized high-efficiency particulate air (HEPA) filters
- Taping off donning/doffing areas at least 2 m from the patient
- Communicating with patients/teams using walkie-talkies or baby monitors
- Establishing ‘clean/cold’ and ‘dirty/hot’ areas of the department with respect to suspected or confirmed COVID-19 patients with dedicated staff in each area
- Universal precautions for all patients. For examples, respirators (e.g. N95, FFP2 or equivalent standard), surgical masks, eye and face protection, water resistant and waterproof gowns, and gloves

Staff who are more at risks such as older adults (e.g. those over 60 years), those with underlying medical conditions (e.g. heart disease, chronic respiratory diseases, cancer), those at risk due to an immunocompromised from a medical condition or treatment (e.g. chemotherapy) and pregnant staff should take care of the lower risk stream of non-COVID-19.

### Perioperative considerations for the COVID-19 virus (16)

OT is potentially a high exposure zones given manipulation of the airway and aerosolisation of respiratory particles, with anaesthesia providers at particularly high risk. It comes with the additional risk inherent in the presence of multiple staff members. The perioperative personnel are at an advantage given their familiarity with maintaining sterility. However, the ancillary staff such as OT cleaners, instrument reprocessing staff and laundry personnel may be at risk. Hence, they should take appropriate precautions and wear PPE (goggles or face shield, surgical mask, heavy duty gloves, long sleeved gown, boots) to avoid exposure to contaminated materials. There are no special decontamination methods other than machine laundering with detergent are required for laundering linens; all surface areas should be disinfected with 0.5% chlorine or 70% alcohol solutions.

### Immediate surgical plan during pandemic (13)

A clear plan should be made to conduct essential operations. Elective operations should be indefinitely postponed to preserve vital resources including hospital beds and PPE. Exceptions are for cancer or highly symptomatic patients and as such the current guidance is not to postpone (Table 1). Each hospital must make a plan based on the current availability of resources. To facilitate decision making and avoid conflicts between patients and providers, a triage algorithm for identification of non-emergent conditions can be used. Surgical emergencies still require prioritisation.

Funding must be adequate to support the hospital and staff with critical surgical services that will continue to be required despite the pandemic response. Consideration and planning to geographically separate COVID+ and non-COVID+ patients should be made. If the case burden is high, consider dedicating one OT to COVID+ operations only. Exposure of health care staff should be reduced as much as practicable. Care pathways and protocols should be developed for COVID+ patients, including the identification of dedicated team members to manage COVID-19 cases each day. Unnecessary patient, their families and health care staff movement through the hospital should be limited as well. Trainees and students, in particular, should not be involved with known COVID-19 positive cases unnecessarily.

Fundamental hygiene practices are essential for everyone. Below are a few suggestions:

- Wash your hands frequently, use alcohol-based hand rub
- Stop shaking hands
- Avoid touching your eyes, nose, and mouth
- Stay home when you are sick. Symptomatic workers should not provide patient care but rather self-isolate
- Cough or sneeze into your elbow (or a tissue, then throw it away)
- Disinfect frequently touched objects and surfaces
- For usual care routines not involving the airway, the use of surgical masks has been associated with decreased transmission and lower rates of health care worker infections and is recommended.

Using a checklist to ensure appropriate precautions are taken for operations with suspected or known COVID-19 infection is also important. Simulation has been also helpful in establishing new routines in the OT. Aerosol-generating procedures that can be provided using other mechanisms should be avoided if at all possible (e.g. metered-dose inhaler instead of nebuliser treatment).

Further planning for the repurposing of OTs to support critical care whilst not precluding the ability to provide lifesaving operative care is needed. Surgical services are already underfunded and poorly prioritised in many health systems, so the commandeering of OTs for use as ICU, which has been proposed in many high-resource settings, must be done with extreme caution. Emergency surgery will still be necessary for obstetrics and to save life (neurotrauma) and limb, and these capacities should not be compromised by taking up all available OT space and ventilators with COVID+ patients. As the average reported time spent on mechanical ventilation has been up to 13 days, critical resources and space will be occupied for many weeks and will be difficult to reclaim once repurposed.

Repurposing of staff for managing COVID-19 cases should be taken into consideration as well. Guidance and training should be provided to make the best use of the technical and clinical skills of all perioperative personnel while protecting them from exposure. Hospitals, professional societies and ministries of health could also provide physician and nursing staff with basic ICU and ventilator management refresher education to improve their capacity to care for COVID-19 patients. Up to date guidelines on COVID-19 management should be provided as knowledge and evidence around best management evolves.

It is also vital to maintain and support staff wellness. It is important to recognise that doctors, nurses, cleaners and other hospital support staff have significant fears and concerns (The fears of transmitting to family or becoming infected oneself, the increase in work hours and the need for childcare coverage) that must be acknowledged and managed. Providers may also be understandably nervous about providing care outside of their normal scope of practice or working beyond their area of competence. Leadership can help manage these by providing information in a transparent way, expressing gratitude for the commitment to patients and colleagues, and reassurances that the system will help protect them and support them and their family.

National CPRC mentioned that severity of the situation and the availability of resources may change on a daily basis. Thus, communication is critical, and an effective communication plan both within and between facilities and health system planners, as well as between providers across the health system and even between countries, is essential and should be established immediately. The preparation of healthcare facilities at large for the safe triage, testing and management of patients with confirmed or suspected COVID-19, and managing surge conditions are needed.

Ethical considerations in resource management are also very important. In many places, the number of ventilators available for persons requiring ventilatory support will be inadequate. In some settings, it is common to reallocate resources from terminal patients or patients with brain death or very low likelihood of recovery (e.g. severe traumatic brain injury) to those with a higher likelihood of recovery. In settings where resources are severely limited and must be rationed, consider creating a committee or utilising standardised risk assessments to determine allocation decisions in advance. This avoids placing the burden of decision making on the frontline health care workers, as these decisions should be not be made ad hoc by the bedside clinician but through careful deliberations by the institution. Cultural and medicolegal context should be taken into account to determine the most appropriate allocation and potential protocols for rationing medical resources and care in advance. Critical testing, PPE, ICU beds, therapeutics, and vaccines should go first to front line health care workers and others who keep critical infrastructure functioning; these workers should be given priority not because they are more worthy but due to their instrumental value in the pandemic response and difficulty of replacing (instrumental value).

### Dedicated Instruments

Should the surgical instrument be reused? (17). Instruments and devices that have been used in procedures for patients with known or suspected COVID-19 should be handled the same as other instruments. Reprocessing should follow manufacturer’s instructions for use (IFU) and be consistent with recommendations in the local infectious disease unit. COVID-19 is an enveloped virus and is susceptible to the Environmental Protection Agency (EPA)-registered disinfectants that are used in the health care setting (18). There are no additional recommendations by CDC for disinfection and sterilisation of these items used for COVID-19 patients.

Instruments should be cleaned, decontaminated, dried and stored in a manner that reduces the risk of exposing patients and personnel to potentially pathogenic microorganisms (19). High-level disinfect or sterilise them according to the manufacturer’s written IFU. They should be packed and stored in individual packs that avoid contamination.

### Dedicated ICU

#### ICU admission in acutely unwell patients (20)

Patients should perform second PCR + Rapid Test prior to ICU admission. If Rapid Test positive, then to discuss with COVID-19 hospital. If accepted, then to transfer to COVID-19 hospital. If not accepted, then send to negative pressure isolation room ICU using designated walkway (only suspected COVID-19) while awaiting second PCR results. If require urgent resuscitation, to transfer to negative pressure isolation room ICU using specific walkway (only suspected COVID-19). If Rapid Test is negative, then to send to negative pressure isolation room ICU using designated walkway (only suspected COVID-19) while awaiting second PCR. If two negative PCR results, then it is considered a non-COVID-19 patient and to be admitted normal ICU cubicle. If PCR is not done on admission or only Rapid Test is done on admission, then a critically ill patient should have 2 PCR done 48 h apart to rule out disease. There is an exception for planned elective admission to ICU after elective surgery. For this group of patients, a negative first PCR is adequate, and the patient is to be admitted to a non-isolation cubicle in ICU.

#### PPE in ICU (21, 22)

Full PPE must be readily available in ICU. Prepacked full PPE sets including Hazmat suit should be put into a bag, to ease donning in the case of emergency. Two sets will be readily available in the emergency trolley with additional sets kept at specific storage area in the ICU together with PAPR. Two nurses to attend with full PPE first. One person to be exclusively in charge to help donning of PPE of medical staff to ensure that all are properly fitted, with Hazmat suit and PAPR if required. PAPR and Hazmat suit will be needed for procedures like intubation and bronchoscopy. Donning and doffing of PPE instructions should be pasted on the wall. Nurses and doctors to undergo training on where and how to don and doff in ICU isolation room, as well as in other non-isolation cubicles. Designated area to doff PPE in non-isolation ICU cubicles/wards to be assigned. Walkie talkie to be available in isolation room for outside communication (to get medications, additional materials). Nurses outside will prepare required materials/medication and put on a trolley, leave it in the airlock to be collected by nurses in the isolation room.

#### Equipment in ICU (13, 18, 20)

Glidescope and ultrasound must be fully covered with plastic when in a room. Ultrasound transducer to be covered with transducer cover if in use. Cleaning should be per biomedic advice. Person cleaning equipment should be made aware of the infectious component and be protected with full PPE. Outside nurse to be fully protected when removing equipment outside isolation room for cleaning. The same ventilator should be used for negative pressure isolation room ICU and should not with ventilator in the other rooms.

#### Procedures in ICU (23)

The viral filter should be on both inspiratory and expiratory ends. Once intubated, to clamp the ETT, remove the viral filter for connection with ventilator tubing. ETCO_2_ monitor i.e. capnograph and closed suction catheter to be connected to patient immediately upon intubation. Disconnection of ventilator should be reduced.

#### Nursing in ICU (23)

Nurses should be divided into COVID-19 team and non-COVID-19 team. The COVID-19 team to take care of COVID-19 suspect or COVID-19 positive patients. The non-COVID-19 team to take care of the non-COVID-19 patients. The COVID-19 team nurses should not be rotated to non-COVID-19 patients. (e.g. patient A is managed by nurse X, Y and Z in three shifts. This team should take care of patient A until second PCR results are available)

#### Decontamination (24)

Container for contaminated PPE for COVID-19 suspect or positive should be clearly labelled for cleaners to take extra precautions. Decontamination of the pathways and negative pressure isolation room ICU used by these patients will be per hospital protocol. The whole ICU need to be close and decontaminated in the presence of a COVID-19 positive case in non-isolation ICU cubicle with no other patients around.

For the presence of a COVID-19 positive case in non-isolation ICU cubicle with existing non-COVID-19 patients in ICU, the whole ICU must be closed and decontaminated plus to consider testing these patients (existing non-COVID-19 patients) and transferring to other hospitals.

#### Visitation in ICU (25)

Potential COVID-19 case in non-isolation room, to limit one visitor/patient. The COVID-19 positive in non-isolation room, then no visitors allowed in ICU. One staff member in front of ICU is stationed to monitor visitor flow in and out of ICU (get visitor’s name and phone number in the case of contact tracing).

#### Contact tracing in the case of COVID-19 positive in non-isolation ICU (26)

In the event of a COVID-19 positive case in non-isolation room in ICU, contact tracing will include, (1) existing non-COVID-19 patients in ICU during that period, (2) all visitors who enter ICU during that period, (3) all ICU staff entering ICU during that period, and (4) all doctors entering ICU during that period.

#### Allocation of ventilators (27)

Triage may not be implemented by a facility without clear sanction from appropriate public health authorities. Systems for sharing information about the number and severity of cases, equipment availability, and staffing shortages could be activated throughout hospital groups and regional networks. Patients are assessed on medical/clinical factors alone, regardless of their work role. The Ontario Health Plan for an Influenza Pandemic (OHPIP) protocol and the sequential organ failure assessment (SOFA) score should be used.

Candidates for extubation during a pandemic would include patients with the highest probability of mortality. Initial assessment using the SOFA scoring system (points based on objective measures of function in six domains: lungs, liver, brain, kidneys, blood clotting, and blood pressure) with best score of 0 and worst score of 24. Time trials assessment at intervals of 48 h and 120 h. Those showing improvement would continue ventilator use until the next assessment, whereas those who no longer met the criteria would lose access to mechanical ventilation.

Chronic care facilities will have to provide more intensive care on-site as part of the general process of expanding care beyond standard locations. The proposed justification for such a strategy would be that more patients could ultimately survive if these ventilators were used by the previously healthy victims of a pandemic. Setting aside the small number of ventilators in chronic care facilities for use by chronically ill people, who likely will have severely limited access to ventilators in acute care facilities.

Clinicians providing direct care will relay data to a supervising clinician serving as a triage officer, who will calculate the SOFA score and make triage decisions but will not provide direct care. Terminal weaning in response to patient preferences can include sedation so that the patient need not experience air hunger. Patients who are extubated against their wishes should be offered appropriate palliative care based on their clinical conditions and preferences. Because transparency is a crucial element of adherence to ethical standards, clinicians must document decisions regarding sedation with extubation. The guidelines do not support the use of manual ventilation devices for patients who do not meet criteria for ventilator access. Daily retrospective review of all triage decisions should be made, to ensure consistency and justice.

Physicians will need to discuss altered standards of care in a disaster, especially for scarce resources such as ventilators. Patients and families must be informed immediately that ventilator support represents a trial of therapy that may not improve the patient’s condition sufficiently and that the ventilator will be removed if the patient does not meet specific criteria. Elective surgeries and other elective procedures that could result in the use of mechanical ventilators should be cancelled (27). Ventilators, supplies, and personnel from ambulatory surgery centres and other facilities not being utilised for COVID-19 patients or not experiencing COVID-19 outbreaks should be transferred.

Anaesthesia ventilation machines capable of providing controlled ventilation or assisted ventilation may be used outside of the traditional use for anaesthetic indication (20, 27).

The ASA and FDA provide specific guidance on how to convert anaesthesia machines for use on COVID-19 patients in respiratory failure (27). Transport ventilators may be used for prolonged ventilation in certain patients. Continuous ventilators labelled for home use may be used in a medical facility setting depending on the features of the ventilator and provided there is appropriate monitoring (as available) of the patient’s condition. Non-invasive ventilation (NIV) Patient Interfaces capable of prescribed breath may be used for patients requiring such ventilator support, including NIV Patient Interfaces labelled for sleep apnoea. Channelling exhalation through a filter is recommended to prevent aerosolisation. Continuous positive airway pressure (CPAP), auto-CPAP and bilevel positive airway pressure (BiPAP or BPAP) machines typically used for treatment of sleep apnoea (either in the home or facility setting) may be used to support patients with respiratory insufficiency. BiPAP may be used for invasive ventilation. If all other alternatives are exhausted, care providers could consider ventilation of two patients on a single ventilator for short-term use, although there are significant limitations to this strategy. Alternatively, manual bag-valve-mask ventilation done by ancillary providers can be considered as a bridging option to mechanical ventilation. The summary of head and neck examination and procedure recommendations can be viewed in Table 4 (28).

#### Management of tracheostomy (28)

In general, most tracheostomy procedures should be avoided or delayed (even beyond 14 days) because of the high infectious risks of the procedure and subsequent care until such time as the acute phase of infection has passed, when the likelihood of recovery is high, and when ventilator weaning has become the primary goal of care. Avoiding early tracheostomy in patients with COVID-19 is suggested because of the higher viral load that may be present at this time. Early tracheostomy was not found to be associated with improved mortality or reduced length of intensive care unit stays in a randomised clinical trial of patients on mechanical ventilation.

Select the patients carefully. If the tracheostomy is assessed as difficult because of anatomy, history, comorbidities or other factors, consider postponing the procedure. Considerations may be given to percutaneous dilatational tracheostomy. Allow it to be done safely with minimal or no bronchoscopy, endotracheal suctioning and disruption of the ventilator circuit. Adequate sedation provided including paralysis to eliminate the risk of coughing during the procedure. Ventilation should be paused (apnoea) at end-expiration when the trachea is entered and any time the ventilation circuit is disconnected. Choose a non-fenestrated, cuffed, tracheostomy tube on the smaller side to make the tracheostomy hole smaller overall (Shiley size 6 for both men and women is adequate). Keep the cuff inflated to limit the spread of virus through the upper airway. Tracheostomy suctioning is performed using a closed suction system with a viral filter. Heat moisture exchanger device is used instead of tracheostomy collar during weaning to prevent virus spread or reinfection of patients. Changing the tracheostomy tube should be delayed until the viral load is as low as possible. Case series of open tracheostomies performed during the severe acute respiratory syndrome (SARS) outbreak can be viewed in Table 5 (29–32).

## Neurosurgical Practices and Decision Making in Times of COVID-19 Crisis: A proposal to Safe and Sustainable Practice in Times of Crisis

### Neurotrauma (33)

Neurotrauma forms the bulk of emergency neurosurgical cases presenting or referred to neurosurgical centres, varying from cases of concussion to severe head injuries requiring urgent surgical intervention. In the current climate of a pandemic crisis, neurotrauma poses various management and logistics issues to the Neurosurgical team in the following aspects as listed.

### Time factor and dynamics of injury

Time between injury to intervention determines outcome. Urgency of intervention outlines management of patient. Ongoing dynamic pathological process in trauma may alter clinical picture between referral to presentation. Pre-emptive management plan is essential to ensure optimum outcome from intervention where feasible.

### Pre-operative screening and risk precaution

Pre-operative screening is vital, particularly those requiring surgical management (34). All cases undergoing surgery poses high risk to operative team due to potential aerosol generating procedure that may occur from intubation/extubation, positioning of patient to prone/park bench position, tracheostomy procedures and prolonged proximity of surgeon to the head region of patient. Ministry of Health Malaysia (MOH) executive recommendation on guidelines for management of surgery during pandemic period (ref KKM 7.60027/14/40) mandates the following (35):

- All cases to be screened with COVID-19 diagnostic tests via reverse transcription-polymerase chain reaction (RT-PCR) test as per protocols
- All procedures and surgeries should be done with full PPE
- Limited essential staff only for surgeries
- Enhanced airborne precaution to be considered when necessary on case by case basis for cases suspected
- PPE protection and precautions to be continued in designated isolation areas in ICU/wards

### Logistic factors (33, 36)

Neurotrauma cases should be managed in a neurosurgical centre. Most neurosurgical departments in Malaysia are situated in major hospitals, often managing high volume of high risk or COVID-19 cases. Principle logistic consideration is to whether trauma cases referred from a non-COVID-19 designated hospital should be managed in COVID-19 designated hospital as this may increase risk of exposure to patients. Alternative options should be taken into consideration where feasible, such as team deployment to manage patients in referring hospitals, training and privileging general surgeons in management of cases and transfer of patients back to referral hospitals for continuity of intensive care management post-operatively.

### Resource availability (37)

Neurotrauma patients require post-operative ventilation with ICU care, often for prolonged duration which may limit availability in case of urgent needs. Ventilators, PPE and ICU are precious commodities in this current pandemic, and fluctuate with time depending on epidemiological dynamics. Available resources are commonly and rightly so; prioritised to patients afflicted by the ongoing pandemic, health care workers (HCWs) involved in their care. Rational balance and anticipation of resource need is essential to optimise usage and sustainable availability in times of crisis.

### Early prognostication (37, 38)

Outcome from neurotrauma depends on various well-defined parameters. Brain damage incurred in primary injury remains irreversible in majority of patients; compounded by secondary factors. Early prognostication is essential in times of crisis for resource allocation. It is paramount to ensure that optimum patient care and outcome remains priority of intention to treat. Determining long term outcome using available prognostic models for decision making in proceeding with active treatment or withdrawing treatment is essential; albeit exceptionally difficult to ensure continuous availability of limited resource. Prognostication based decision is best made with team consensus using all available scientific evidence present.

### Proposed Recommendations for Management of Brain Trauma (6)

All brain trauma management is in accordance with the Brain Trauma Foundation (BTF) guidelines recommendations. All neurosurgical emergencies must be referred to the respective neurosurgical team for consultation and management plans (Table 6) (39).

Brain trauma requiring urgent surgical intervention (decompression) with or without intensive care monitoring is best managed in hospitals with dedicated neurosurgical facilities or available neurosurgical services (Level II). Brain trauma not requiring urgent decompressive surgical intervention, but which may require or benefit from intracranial pressure (ICP) monitoring and intensive care management (Level IIB) is best managed in centres with available resources to provide objective assessment and management plan, reduce the duration of ICU stay and intensive management and early weaning from intensive therapy. Brain trauma not requiring urgent decompressive surgical intervention, but which may benefit from ICP monitoring and intensive care management in situations where resource availability is limited may be managed with cerebral perfusion pressure (CPP) based target therapy (Level III) and serial CT scan at 6 h–12 h intervals in an intensive care setting where feasible.

Brain trauma requiring surgical intervention but with limited resources available at dedicated neurosurgical facilities; the following may be considered (anecdotal evidence based on local/regional practice):

- Deployment of neurosurgical team to primary referral hospital where feasible to facilitate timely intervention
- Surgical intervention in neurosurgical facilities with subsequent transfer back to primary referring hospitals for continuity of intensive care management

Brain trauma not requiring any surgical intervention but requiring close observation is best managed in dedicated neurosurgical centres or available neurosurgical services if the risk of potential deterioration is deemed to be high (e.g. burst temporal/frontal lobes) and duration and distance of transfer may result in a delay of treatment. Brain trauma requiring multidisciplinary management is best transferred and managed in dedicated neurosurgical and multidisciplinary facilities (Level II). Patient ideally should be managed in high dependency unit or acute cubicles. If the risk of potential deterioration from progression of injury is not deemed high risk, then the patient is best managed in primary referral hospital with expectant medical management and observation with frequent neurosurgical consult and input. Early clinic appointment upon discharge for review where feasible.

### Screening and Risk Precautions for Pandemic COVID-19 Exposure (40)

**Figure 2:**
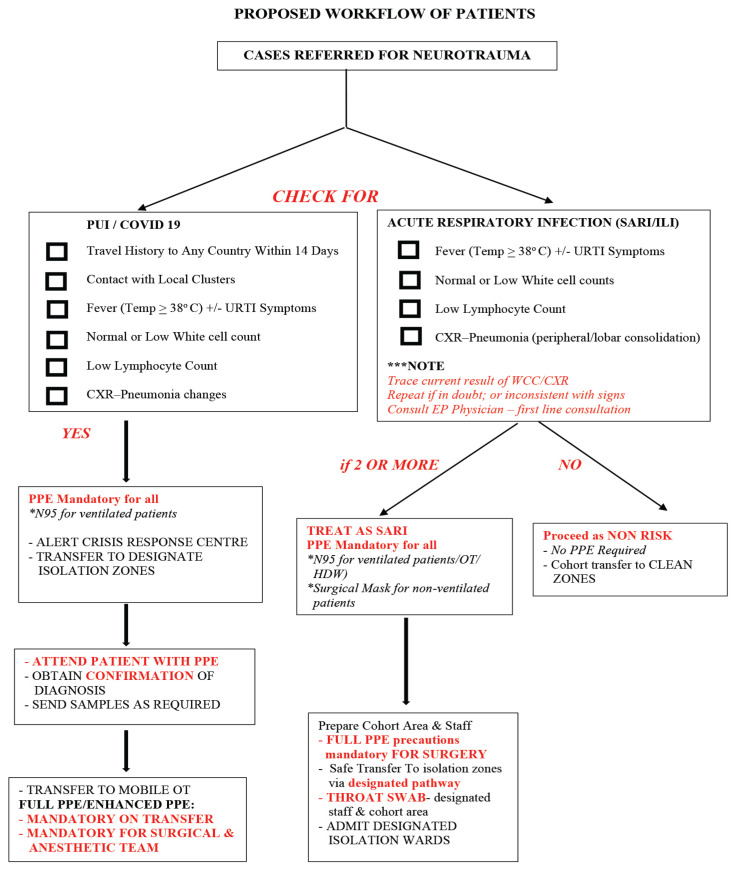
Proposed workflow of patients referred for neurotrauma

**Figure 3:**
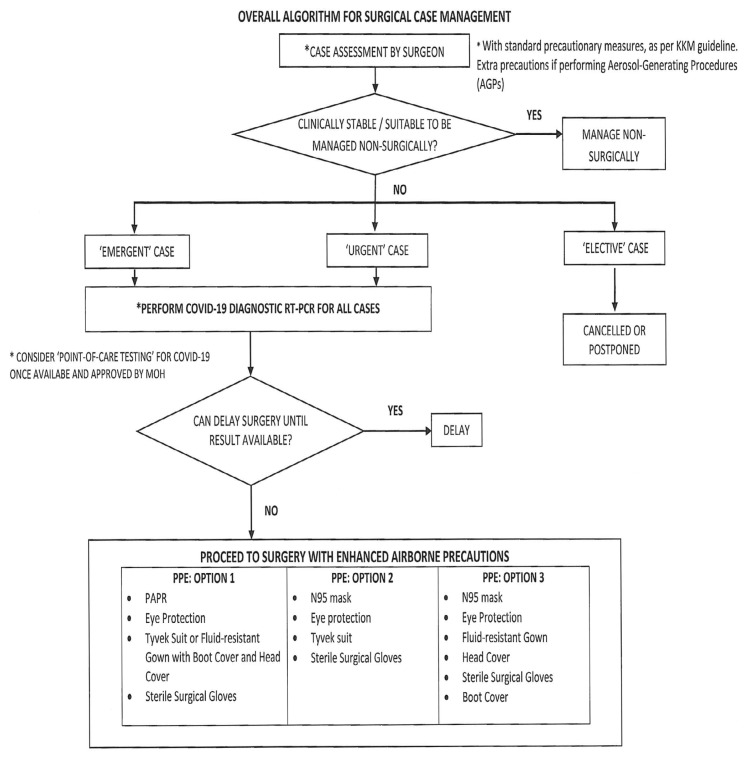
Algorithm for surgical case management during COVID-19 outbreak

Screening for traumatic brain injury cases is strongly recommended for safety of HCWs. Screening recommendations are in accordance with MOH guidelines (Figure 2). Risk of COVID-19/PUI/severe acute respiratory infections (SARI) should be ruled out as per MOH guidelines and hospital protocols prior to transfer of cases for further management (Figure 3). Secondary screening should be done by attending neurosurgical team on arrival. Relatives must accompany for confirmation of history of potential exposure as per MOH protocol and directives. Confounding factors must be taken into consideration during screening, including: i) potential aspiration in patients with low Glasgow coma scale (GCS); ii) metabolic response resulting in abnormal white blood cell count (WCC) and elevated temperature; iii) co-existing chest injuries; and iv) post-intubation changes on chest radiograph (CXR) common.

Note: All ventilated cases pose a high risk of aerosol exposure to HCWs. It is vital to be meticulous and vigilant for potential risk:

- consult ID/medical team and screening done for all cases undergoing surgery
- CT thorax in selected high-risk suspicious cases is recommended
- potential high-risk cases must be managed in dedicated wards/units as per hospital protocols to avoid contamination and exposure of clean areas and staff
- consideration to managing potential high risk/or high risk in COVID-19 designated hospitals via team deployment if neurosurgical centre located at non-COVID-19 hospital
- full PPE practice is recommended in all potential or high risk SARI cases until confirmation is obtained

### Rationale for Early Prognostication and Resource Allocation (41, 42)

Critical resources for optimum neurosurgical services remain limited and may continue to fluctuate in time of crisis. These include ventilators, PPE, ICU availability and operative instruments. The rationale for early prognostication is recommended for optimum resource usage and allocation to ensure beneficial outcome and sustainable supply. Prognostication is based on available scientific evidence to guide in management options and rationale of resource allocation. Early triage is required for timely and appropriate treatment and enables surgeons to prioritise management according to available resources and the potential outcome. This will help in limiting the proportion of patients in a vegetative state and limiting burden to family and available resources at the time of crisis. In the end, it will help to prepare family with a realistic outlook on the potential outcome.

Prognostic factors include: i) age > 60; ii) GCS post-resuscitation: motor score M2 — poor outcome; iii) pupils-bilateral fixed/dilated pupils; iv) systolic blood pressure < 90 mmHg — sustainable/multiple episodes; and v) Marshall CT grade. Age, GCS motor score and pupillary changes are the three main prognostic factors determining the outcome. The caveats to prognosticating outcome include:

- accurate assessment of GCS score and pupillary size post-resuscitation
- adequate/optimum resuscitation measures
- antecedent injuries or extracranial factors accounted for and resuscitation/correction done
- adequate reversal of sedative agents
- trial of mannitol/hyperosmolar therapy

## Emergency Vascular Cases: Management of Neurovascular Emergencies During COVID-19 Pandemic Crisis (43)

Neurovascular emergencies constitute the third most common reason for emergency hospital admission after neurotrauma and strokes. Typically, neurovascular emergencies are life threatening and warrant urgent treatment on presentation. These include presentations of aneurysmal subarachnoid haemorrhage (SAH), ruptured arteriovenous malformation and ruptured dural arteriovenous fistula.

### SAH

**Figure 4:**
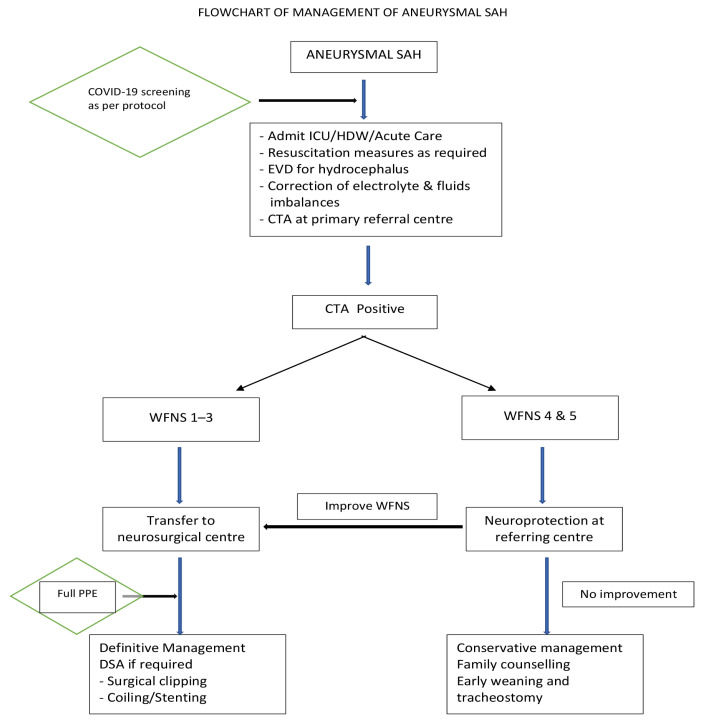
Proposed workflow of patients referred for SAH

Aneurysmal SAH is a devastating clinical entity. The natural history of aneurysmal SAH remains unfavourable with a cumulative re-rupture rate at 50% at 2 weeks after initial presentation and overall mortality of 60%. Prognostication of aneurysmal SAH is well defined according to the World Federation of Neurosurgery (WFNS) grade system predictive of outcome following aneurysmal rupture and Hunt & Hess grade which predicts mortality rate in patients. Management of aneurysmal SAH during times of crisis must be guided by expected prognosis as defined using these grading systems. This is essential in times of pandemic crisis as difficult decisions need to be made to preserve life and function in face of limited and precious resources essential to the management of patients (41). The proposed recommendations can be viewed in Figure 4.

### Aneurysmal SAH (44)

Adequate resuscitation measures should be instituted on admission where required. These include the airway, breathing and circulation (ABC) and mechanical ventilatory support as required with correction of fluid and electrolyte abnormalities if present at primary referral centres. The physician receiving the referral should get an accurate assessment of WFNS, and Hunt & Hess grades on presentation. If hydrocephalus is present on ictus, urgent ventricular drainage should be performed at centres with neurosurgical facilities or trained and privileged surgeons at the designated non neurosurgical centres. A noncontrast CT brain and CT angiogram (CTA) are the first line investigative parameter at the admitting hospital before transfer to a centre with neurosurgical facilities or services.

### CTA negative SAH

When perimesencephalic SAH is the likely diagnosis after confirmation by a neuroradiologist, then the patient should be managed expectantly. There will be no further imaging required. However, if aneurysmal rupture cannot be ruled out, then the recommendation is based on the WFNS grading. For WFNS 1–3, a digital subtraction angiography (DSA) is recommended at the primary centre if available or transfer to centres with available facilities and neurosurgical services. For WFNS 4 and 5, the patient should stay at the primary centre if feasible with a repeat CTA in 1 week. A continuous neurological assessment should be documented and if whenever an improvement is noted, then the patient should be considered for transfer.

Poor grade aneurysm cases may benefit from continued neuroprotection. However, multiple factors should be taken into accounts such as age, comorbidities and available resources to sustain prolonged care in such patients. The decision to consider conservative management if no further improvement in WFNS score is the prerogative of attending consultants.

### CTA positive SAH - WFNS grade 1–3

The quality of CTA is important in determining the next treatment of care. For patients with WFNS Grade 1–3, if CTA deemed adequate by attending consultants for safe and effective definitive management by surgical clipping, then the recommendation is to proceed for surgical treatment as urgently possible at the centre with available neurosurgical services. If CTA deemed inadequate for definitive management, then the patient should be transferred for DSA at centres with available radiological and neurosurgical services. The choice of treatment between surgical treatment and endovascular treatments should remain similar to current standards. However, interventional neurovascular services in Malaysia are limited to a few major hospitals. Transferring patients across states in times of limited resources may result in unnecessary delays and worsening outcomes from potential deterioration during the interim period. Thus, various factors should be taken into consideration when deciding the best treatment options in times of crisis. They include:

- location, morphology and anatomical complexity of aneurysm and parent vessel – anterior circulation vs posterior circulation
- presence of clots causing potential deterioration
- availability of essential equipment – clip types and options
- trained and experienced personnel to undertake the procedure
- risk of exposure in suspected cases to health care personnel related to the duration of surgery
- access and availability of endovascular services and facilities
- patient decision after thorough explanation and discussion

If DSA is required, the option of definitive management of endovascular coiling at the same setting should be considered. There are several reasons which include: i) minimising the risk of aerosol disbursement that is highest during intubation/extubation; ii) early definitive management can be achieved at the same setting with reduced risk of re-rupture; iii) treatment of vasospasm if present for applicable cases done at the same setting; and iv) reducing the risk of exposure to personnel from a second GA procedure.

### CTA positive SAH - WFNS grade 4 and 5

Patients with WFNS grade 4 may benefit from neuroprotective measures. Definitive management should be considered in selected cases such as: i) young age; ii) no morbidities/co-morbidities; and iii) choice of treatment depends on available services and following discussion between attending consultant and family. For patients with WFNS grade 5, conservative management should be considered if no further improvement achieved following a period of neuroprotection. The decision is made through a collective discussion between attending physician and family members.

### Aneurysms without SAH

A thorough history should be elicited to determine any evidence of sentinel haemorrhage that may appear trivial to patients. An appropriate diagnostic modality is required to look for radiological evidence of recent i.e ‘teat sign’. Within an applicable timeframe, a lumbar puncture should be considered. An aneurysm with neurological symptoms and signs should be planned and treated in a timely manner as a semi-emergency case; an example is a patient with posterior communicating artery aneurysm presenting with ptosis, rather than a delayed surgery in this current pandemic of uncertain duration to avoid potential irreversible neurological compromise or worsening deterioration. In cases of multiple aneurysms, the treatment should be undertaken for ruptured aneurysm as well as aneurysm with increased risk of rupture, if deemed feasible at the same sitting.

### Arteriovenous Malformations

Arteriovenous malformations (AVMs) are a heterogeneous group of neurovascular abnormalities with an incidence of 1.1/100000 population. Anatomically AVMs are defined as a complex of abnormal arteries and veins that communicate directly without an intervening capillary bed. AVM presents with haemorrhage in as many as 50% of cases. The natural history of AVMs is more favourable as compared to aneurysmal SAH, with an annual rupture rate of 4%, and recurrent haemorrhage rate of 6%–17% in the first year following a rupture (45).

Cases of AVM presenting with haemorrhage may require urgent surgical management. The proposed recommendations are divided into three categories: i) AVM rupture with mass effect; ii) AVM rupture without mass effect; and iii) AVMs not presenting with haemorrhage (46–48).

### AVM rupture with mass effect

Any ruptured AVM with mass effect should be transferred to a centre with neurosurgical services or facilities. A CTA should be performed on admission to confirm the diagnosis of ruptured AVM and to determine the location of AVM in relation to clot. This will aid a safe surgical access planning. Following an urgent surgical evacuation of a clot, the patient should be managed in an intensive care setting for neuroprotection. A post-operative DSA/CTA is warranted to look for potential high risk factors for haemorrhage, such as nidal aneurysm/varix and to consider an endovascular treatment if feasible and required. Definitive treatment should be deferred safely to a later date if feasible.

### AVM rupture without mass effect

The patient should be transferred to a centre with neurosurgical facilities, particularly when AVM presents with intraventricular haemorrhage (IVH) or CTA shows evidence of nidal aneurysm or varix. If no IVH present and CTA shows no high risk factors of haemorrhage, the patient may be managed at a non neurosurgical centre with early follow up scheduled in the clinic for review.

### AVMs not presenting with haemorrhage

The management is mainly medical treatment to optimise seizure control or headache. All definitive treatment should be deferred to a later date.

### Dural AV Fistulas (49)

These are rare conditions that comprise of fistulas connecting branches of dural arteries to dural veins or venous sinuses. Dural arteriovenous fistulas (DAVFs) are typically stable lesions with a reported annual haemorrhage risk ranging between 6%–8% (10), and mortality rate ranges at 6%–20%. Endovascular modalities remain the diagnostic and therapeutic modality of choice. The recommendations are proposed for the management of (1) ruptured cranial DAVF and ruptured spinal DAVF. Patients who presented with ruptured cranial DAVF should be transferred to a neurosurgical centre with endovascular facilities for urgent surgical management of clot with mass effect, if present, and definitive endovascular management. Patients who presented with ruptured spinal DAVF requires transfer to a neurosurgical centre with endovascular facilities for urgent treatment, especially when there is rapid neurological deterioration.

## Spontaneous Hypertensive Haemorrhage

Some patients presented with spontaneous hypertensive haemorrhage that may or may not require surgical intervention. Patients that are not a candidate for surgical interventions include: i) small, deep haemorrhage; ii) large haemorrhage without hydrocephalus, IVH or neurological deterioration; and iii) those with supratentorial haemorrhage with a GCS score below 8 unless this is because of hydrocephalus (50). A poor prognosis is expected in the following conditions: ii) candidate with GCS score is 5 or less; and ii) the haematoma is very large and death is expected (50). In this condition, a careful consideration should be taken and a family meeting should be done sooner.

Surgical interventions are recommended in the following categories:

- patients with intracranial haemorrhage who develop hydrocephalus is a candidate for external ventricular haemorrhage (50)
- patients that require supratentorial hematoma evacuation include: i) deteriorating patients where evacuation of haematoma might be considered as a life-saving measure (Class IIb; Level of Evidence C); ii) early hematoma evacuation is not clearly beneficial compared with hematoma evacuation when patients deteriorate (Class IIb; Level of Evidence A); and iii) the effectiveness of minimally invasive clot evacuation with stereotactic or endoscopic aspiration with or without thrombolytic usage is uncertain (Class IIb; Level of Evidence B) (51)
- a decompressive craniectomy (DC) with or without hematoma evacuation (51) might reduce mortality for patients with supratentorial ICH who are in a coma, have large hematomas with significant midline shift, or have elevated ICP refractory to medical management (Class IIb; Level of Evidence C)
- in infratentorial hematoma evacuation (51), patients with cerebellar haemorrhage who are deteriorating neurologically or who have brainstem compression and/or hydrocephalus from ventricular obstruction should undergo surgical removal of the haemorrhage as soon as possible (Class I; Level of Evidence B). Initial treatment of these patients with ventricular drainage rather than surgical evacuation is not recommended (Class III; Level of Evidence C)

## Middle Cerebral Artery (MCA) Infarction

The diagnosis of middle cerebral artery (MCA) infarction depends on the clinical presentation, neurological findings, followed by radiological imaging. Patients with suspected transient ischaemic attack (TIA) should be assessed by a specialist physician before a decision on brain imaging is made, except when haemorrhage requires exclusion in patients taking an anticoagulant or with a bleeding disorder when noncontrast CT should be performed urgently (50). Further imaging, such as carotid imaging is essential for any patient presenting with symptoms suggesting of an anterior circulation cerebral ischaemia who might be suitable for intervention for carotid stenosis. Patients with TIA or acute non-disabling stroke with stable neurological symptoms who have symptomatic severe carotid stenosis of 50%–99% (NASCET method) should receive an urgent carotid endarterectomy (within 7 days). The treatment tends to happen in a vascular surgical centre routinely participating in national audit (50).

The indications for mechanical thrombectomy (MT) (50) are when there are proximal large artery occlusion as an adjunct to intravenous thrombolysis (IVT), and for those patients with contraindications to IVT but not to MT. Another indication is when major vessel occlusion is in the posterior circulation, up to 24 h from known onset.

**Figure 5:**
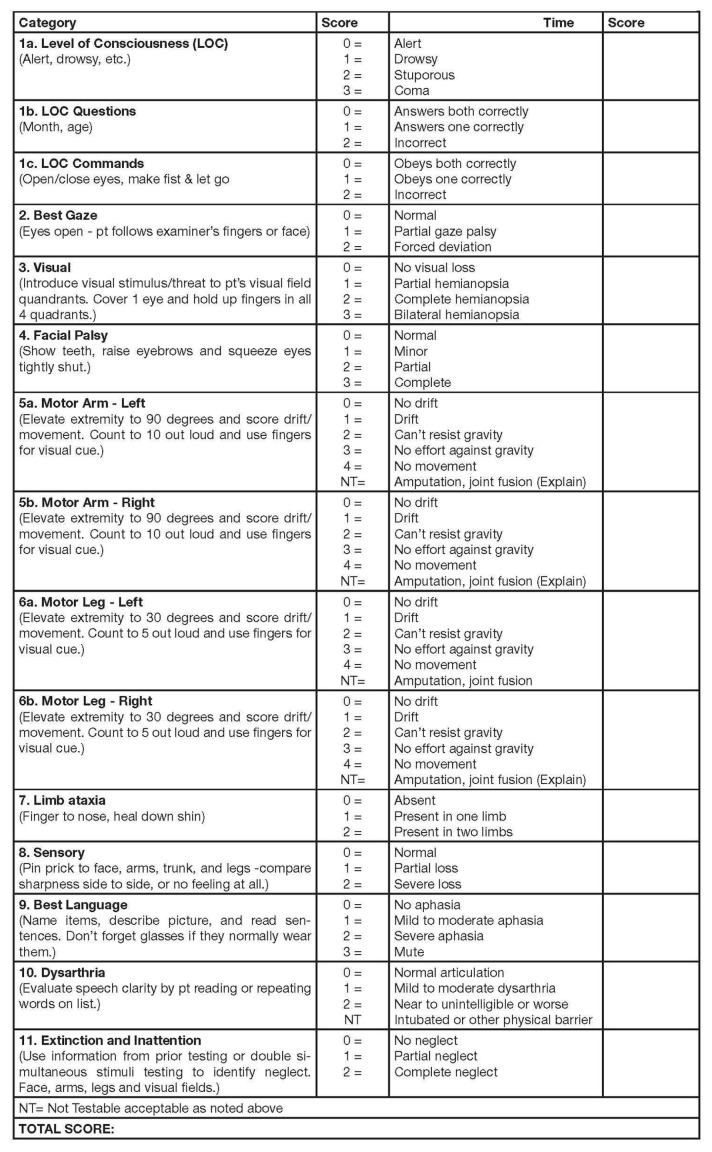
The National Institutes of Health Stroke Scale quantifies stroke severity

The indications for decompressive hemicraniectomy are as follows (50):

- treated within 48 h of stroke onset
- pre-stroke modified Rankin Scale score of less than 2
- clinical deficits indicating infarction in the territory of the MCA
- National Institutes of Health Stroke Scale (NIHSS) (52) score of more than 15 (Figure 5)
- a decrease in the level of consciousness to a score of 1 or more on item 1a of the NIHSS
- signs on CT of an infarct of at least 50% of the MCA territory with or without additional infarction in the territory of the anterior or posterior cerebral artery on the same side or infarct volume greater than 145 cubic centimetres on diffusion-weighted MRI

Whereas, in cerebellar infarction (53), ventriculostomy is recommended in the treatment of obstructive hydrocephalus. Concomitant or subsequent decompressive craniectomy may or may not be necessary on the basis of factors such as: i) the size of the infarction; ii) neurological condition; iii) degree of brainstem compression; and iv) effectiveness of medical management. There are times when an emergency carotid endarterectomy (CEA)/carotid angioplasty and stenting will be useful as clinical indicators or brain imaging suggests (53). An example is when a small infarct core with large territory at risk (e.g. penumbra), compromised by inadequate flow from critical carotid stenosis or occlusion. However, in patients with unstable neurological status (e.g. stroke-in-evolution), the efficacy of emergency or urgent CEA/carotid angioplasty and stenting is not well established.

### Temporary Emergency Guidance to Stroke Centres During the COVID-19 Pandemic (54)

Stroke complicated COVID-19 infection in 5.9% of patients at a median 10 days after symptom onset. Stroke mechanisms may vary and could include hypercoagulability from critical illness and cardioembolism from virus-related cardiac injury. The clinical presentation in stroke patients typically manifested as CNS involvements. The most common neurological manifestations in COVID-19 patients were dizziness (16.8%), headache (13.1%) and encephalopathy (2.8%). The most common peripheral signs and symptoms were anosmia (5.1%), dysgeusia (5.6%) and muscle injury (10.1%, detected by elevated creatine kinase). Patients with stroke were older, had more cardiovascular comorbidities, and more severe pneumonia.

## Personnel Protection Requirements When Dealing with Stroke Patients

Ideally, every stroke patient would be treated as potentially infected, hence the requirement of PPE. Many teams have begun using telemedicine both within their own ED and regionally. This solution avoids the use of needed PPE, allows a reasonable stroke evaluation, avoids unnecessary interfacility transfers, and reduces exposure risk for the stroke team. In the setting of the pandemic, full compliance to clinical practice guidelines has become a goal, not an expectation. Each team must use their judgement, guided by local realities, and continue to try to treat as many acute stroke patients as possible.

Patients with large intracerebral haemorrhages, SAH or large ischemic strokes at risk for herniation must be monitored in an intensive care setting with appropriately trained personnel, where possible. Appropriate resource should be allocated for critically ill stroke patients. Appropriate intensive care of these seriously ill patients with haemorrhagic stroke, some of whom are also young and with an excellent long term outcome, should be maintained. However, in each locality, specialists from all intensive care specialities e.g. pulmonary, cardiology, neurology, neurosurgery must discuss the relative merits of prolonged ICU care for any particular patient.

### Challenges and Potential Solutions of Stroke Care (55)

The establishment of stroke networks and care systems can deliver a high quality emergency stroke care at all times, particularly at times of crisis. Although there is a strong case for such centres to be the system of care, it is particularly important to have services that can continue to function. The hospital should inform the emergency medical system and the public that these centres will be protected and will remain fully operational even during crises. The hospital or stroke team should regularly update and educates the health professionals and the public, especially those who are at high risk of stroke to recognise a stroke and call emergency medical services immediately. Those patients should be taken to one of the designated stroke centres to avoid significant delay in transferring patient from one hospital to the other.

## Elective Neurosurgery

**Figure 6:**
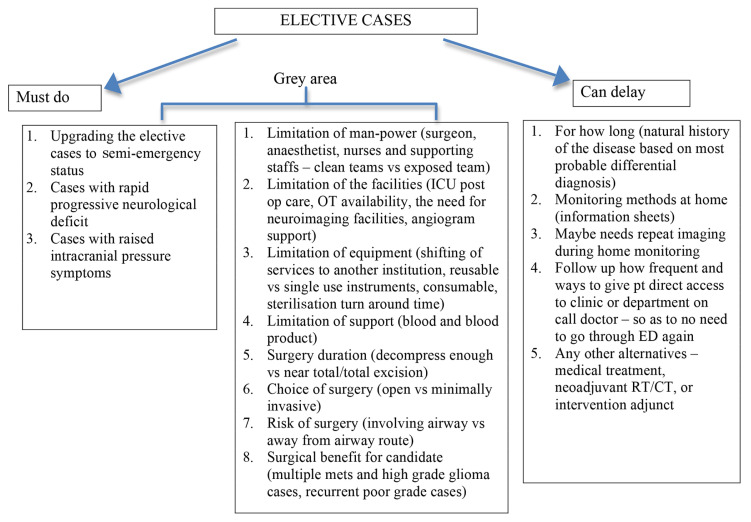
Availability of manpower and equipment for surgery

Categorising elective neurosurgical cases at a time of COVID-19 pandemic is adapted and as per guidelines (with minimal modification) — Perioperative Mortality Review (POMR): Prioritisation of Cases for Emergency and Elective Surgery (2nd Revision) (56) (Table 7). The tier status of each case is according to the urgency and the decision will be based on: i) natural history of the disease; ii) patient’s neurological status; and iii) availability of manpower and equipment’s for surgery (Figure 6).

To summarised, all elective neurosurgery should be postponed (1, 21). In patients with suspected COVID-19, the surgery should be deferred for at least 14 days, with an appropriate test taken to confirm the status (1, 21). The elective urgent inpatient diagnostic and surgical procedures should be shifted to outpatient settings, when feasible (2).

## Safety Precautions During Surgery

### Dangers During Neurosurgical Procedure (2, 18, 19, 24, 28, 57)

According to limited data from CDC, COVID-19 has been detected in blood specimens and it is unknown whether the virus is viable or infectious in extrapulmonary (outside the lungs) specimens. There have been some reports that COVID-19 is present in stool and maybe transmissible through the faecal-oral route. Bronchoscopy, tracheostomy and thoracic cases may have a higher risk for airborne transmission of COVID-19 because the nature of the procedures involves the respiratory tract, which could lead to aerosolisation of the virus. Procedures that may aerosolise blood and body fluids during surgery include: i) electrocautery of blood or tissue; ii) laparoscopy; iii) endoscopy; iv) use of intraoperative debridement devices with irrigation (e.g. hydrosurgery, pulse lavage or low frequency ultrasonic debridement); and v) use of high speed powered equipment (e.g. saws and drills).

Surgical smoke represents another important issue to tackle during surgery. It is recommended for the evacuation of all surgical smoke as it contains hazardous chemicals, ultra-fine particles, viruses, bacteria and cancer cells. The earliest detected case of COVID-19 was in China on 17 November 2019. As such, there is currently no research on the transmission of the virus through surgical smoke. However, there is no indication or proof that COVID-19 is not transmissible through surgical smoke. Research studies have demonstrated the presence of viruses (e.g. human papillomavirus) in surgical smoke with documented transmission to health care providers. According to limited data from the CDC, SARS-CoV-2 RNA has been detected in blood specimens and it is unknown whether the virus is viable or infectious in extrapulmonary (outside the lungs) specimens. In similar coronaviruses, viable and infectious SARS-CoV was isolated from blood specimens, although infectious MERS-CoV was only isolated from the respiratory tract.

Of importance to neurosurgeons, the use of high speed drills and also electrocautery during surgery will cause aerosolised blood and body fluid. Thus, increasing exposure of neurosurgeons to the virus. However, the risk of transmission of COVID-19 through aerosolised blood and body fluids is unknown. Thus, extra precautionary measures must be taken during procedures for protection. Proper PPE must be worn during any neurosurgical procedure to prevent transmission.

### RA Versus GA in Neurosurgery (58, 59)

COVID-19 was declared a pandemic by WHO on 11 March 2020 because of its rapid worldwide spread. COVID-19 has achieved effective and sustained human-to-human transmission via contact, droplet and likely airborne routes. As with previous outbreaks such as severe acute respiratory syndrome (SARS), influenza A (H1N1) 2009 infection and the Middle East Respiratory Syndrome, this would require heightened precautions and tailoring our anaesthetic practice to reduce exposure to patients’ respiratory secretions and the risk of perioperative viral transmission to healthcare workers and other patients. In particular, this should involve minimising the many aerosol-generating procedures we perform during GA, such as bag mask ventilation, open airway suctioning and endotracheal intubation. During the SARS outbreak, intubation was one of the independent risk factors for super-spreading nosocomial outbreaks affecting many healthcare workers in Hong Kong and Guangzhou, China. Nevertheless, to avoid any airway manipulation, the use of RA techniques (e.g. peripheral nerve blocks and/or central neuraxial blocks) may be preferred. Thus, RA manipulation should be considered whenever surgery is planned for a suspected or confirmed COVID-19 patient or any patient who poses an infection risk. RA has benefits of preservation of respiratory function, avoidance of aerosolisation and hence viral transmission.

There is no proper guideline and recommendation as of today regarding the use of RA in COVID-19 patients. However, general precautions and PPE should be applied for all the healthcare workers even though the patient is undergoing RA. This is because, in case of failed RA, GA must be used for the surgery. Anaesthesia providers for these patients should be well-versed in both GA and RA techniques. For neurosurgery patients, RA such as scalp block must be considered in simple procedures such as borehole. For emergency neurosurgery cases, most of it would be involving patients with poor GCS thus making RA not as feasible as the patient would be already intubated.

## The Screening for Neurosurgical Patients

COVID-19 is an infectious disease introduced to humans for the first time. Individuals can be infected by breathing in the virus within 1 metre of a person who has COVID-19, or by touching a contaminated surface and then touching their own mouth, nose, or possibly their eyes. On 30 January 2020, WHO declared the outbreak as a Public Health Emergency of International Concern (PHEIC), and by 11 March 2020, the outbreak has rapidly accelerated to become pandemic. Until 3 April 2020, there were 976, 249 confirmed cases, with 50,489 deaths, affecting 207 countries, including Malaysia (60).

Following the pandemic of COVID-19, there is a major shift of practices among surgical departments in response to an unprecedented surge in reducing the transmission of disease. With pooling and outsourcing of more HCWs to emergency room, public health care services and medical services, further in hospital resources are prioritised to those in need. Along with slowing and breaking the transmission of COVID-19 by social distancing, the neurosurgical outpatient clinic, elective and non-emergency surgery are delayed. This will reduce the face-to-face contact with potential COVID-19 cases, and shields patients and HCWs from the virus.

Every neurosurgical team has to re-evaluate the timing of operation in those patients with neurosurgical disease that are in need of treatment. The real risk of proceeding and the real risk of delay should be carefully assessed. When considering a delay in treatment to a time where COVID-19 is less prevalent, the decision making process must always take into account each patient’s courses of disease, social circumstances and needs. It is imperative to balance the requirements of caring for COVID-19 patients with imminent risk of delay to others who need care (61).

## Screening Process for Neurosurgical Patients

### Patient under investigation

Currently, patient’s screening process is crucial. The MOH recommendations of screening involve questionnaires to identify suspected patients (1, 62). Patients who meet certain criteria should be evaluated as a patient under investigation (PUI). These general questions to all patients include:

- Do you have any fever or acute respiratory infection (sudden onset of respiratory infection with at least one of: shortness of breath, cough or sore throat)?
- Do you have any history of travelling to or residing in affected countries in the past 14 days?
- Did you have any contact with a confirmed COVID-19 case within the past 14 days?

### Laboratory tests

There are two laboratory tests that can be used to detect COVID-19 (1, 61):

- RT-PCR. The sample commonly taken is upper airway specimens (pharyngeal swabs, nasal swabs, nasopharyngeal secretions). Among patients with confirmed positive in respiratory tract, ~ 50%–60% of patients have detected viral load in faeces, ~30%–40% in the blood, while the lowest positive rate is in urine samples.
- Rapid Test kit (RTK) serology for serum antibody IgM and/or IgG. This can be used as diagnostic criteria for suspected patients with negative PCR detection. During follow-up monitoring, IgM is detectable 10 days after symptom onset and IgG is detectable 12 days after symptom onset. The viral load gradually decreases with the increase of serum antibody levels.

Urgent/emergent cases are previously defined as patients requiring access to surgical treatment within 24 h of the decision to operate (63). However, with the current pandemic, access to surgical treatment may be delayed and some patients could face increasing morbidity/mortality by the time surgery happens. Other countries have now come up with guidelines for the triage, or ranking in order of priority, of surgical patients. The American College of Surgeons (ACS) describes the acuity scales based on Tier classification, with most cancers and highly symptomatic patients considered Tier 3a (do not postpone) (64). Other than trauma and life threatening condition, other treatments are recommended to postpone, if possible. Then what will happen to all elective cases that will be pushed back for a further few months? Will that add to the mounting burden of long waiting lists that is already stretched?

**Figure 7:**
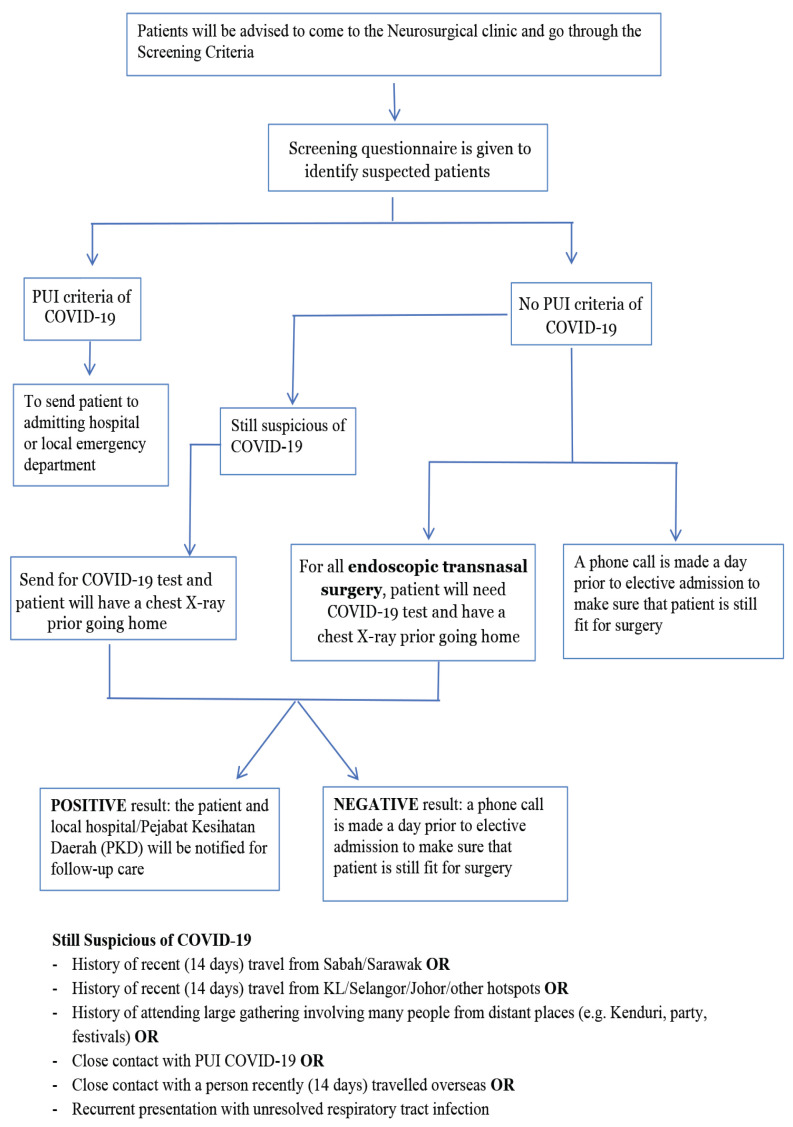
Proposed workflow of patients during neurosurgical outpatient clinic

To delineate the current situation, we propose a few steps that will in future, limit and protect both patients and surgeons from the risk of transmission (Figure 7). Special consideration is given to pre-operative patients needing endoscopic transnasal surgery, even for asymptomatic patients (8, 57).

## PPE for Neurosurgical Procedures

The transmission of COVID-19 is predominantly via respiratory droplets (e.g. coughing and sneezing) and contact with contaminated surfaces (18, 60, 65). However, earlier studies have shown the presence of the virus in conjunctival secretions and even stool (65). Hence all body fluids except for sweat should be considered as potentially infectious (66). Contamination of the surrounding environment may also occur following aerosol-generating procedures (AGP) which include the use of high-speed devices or when splashing or spillage of bodily fluid is expected (66). Appropriate steps especially in these environments need to be taken to disrupt the transmission of COVID-19 and reduce risk of infection. These environments are considered as high-risk environments and include the ICU, high-dependency unit (HDU) and OTs. The emergency rhesus areas where suspected or confirmed cases of COVID-19 are managed are also considered as high-risk areas (66). All high-risk areas require level III PPE (Table 8) (66).

### General Guide for PPE

PPE is only a part of the safe system of working (67). Clinical staff must be trained and competent in the use of PPE in their respective hospitals. Table 8 shows the recommended PPE for clinical setting (15, 61, 68). PPE should be located close to the point of use and should be stored in a clean and dry area to prevent contamination. Ideally, PPE is for single use and changed between patients unless in a situation of PPE shortage or when re-usable PPE is used. The used PPE must be disposed of in designated waste streams. The practice of donning (putting on) and doffing (taking off) of PPE must be done in designated areas safe for the respective procedures (18, 24).

**Figure 8:**
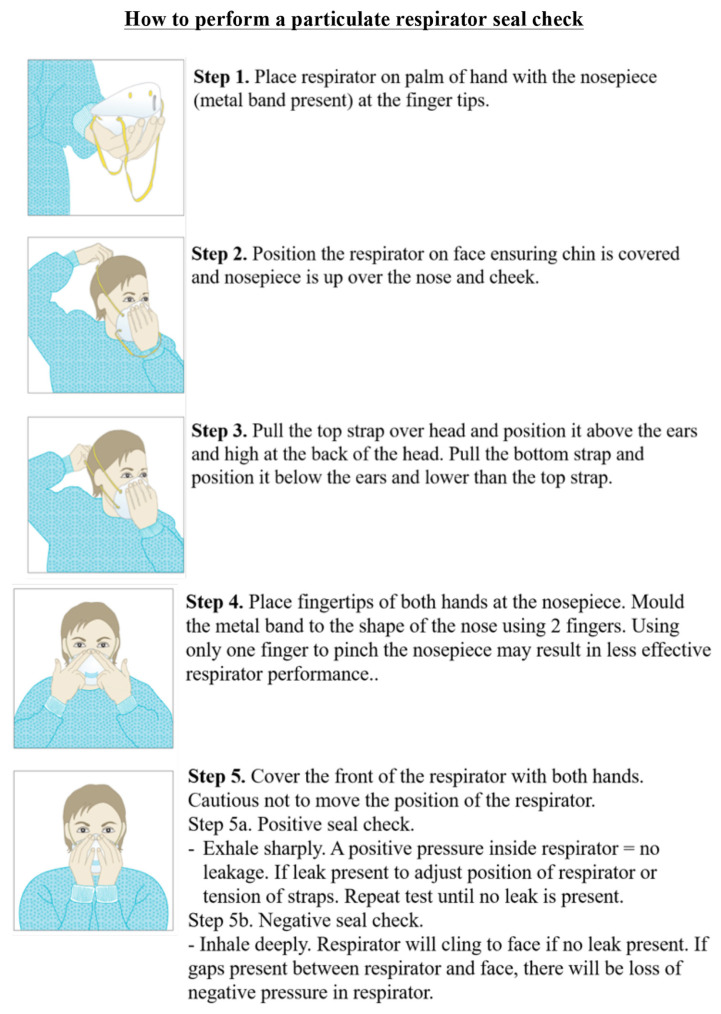
How to perform a particulate respirator seal check

Different hospitals may have different arrangements of clinical areas (OT, wards, clinics, etc.). Hence, the physicians must make sure to be aware of the designated areas for donning and doffing prior to putting on PPE. Ideally, each clinical staff donning must be supervised by another competent clinical staff especially to assist in donning and to make a final visual inspection of PPE. Recommended PPE components:

- Protective medical gown should be long in length, long sleeves, rear-fastening and fluid-resistant. Also, include protective, fluid-resistant boots with disposable fluid-resistant covers when appropriate (high-risk procedures). For the gloves, the recommendation is to use disposable, non-sterile latex gloves for non-sterile procedures, whereas for sterile procedures is to use disposable sterile latex gloves. Double gloving is essential in those procedures.
- For the face/eye protector, ideally a disposable visor which covers the whole face including chin will give better protection than glasses and contact lenses.
- For the respirator/mask, PAPR is recommended and preferred for high-risk procedures including AGP. A minimum of N95 mask with face protector (i.e. visor) for AGP (28, 69–71). For SARS-CoV, there is limited evidence (from observational studies) showing a protective effect of up to 80% of N95 masks (equivalent to FFP2 masks) used by healthcare workers. Hence, although the CDC recommends N95 masks or higher level respirators for AGP, N95 respirators are not recommended for AGP in the UK (19, 69). The masks used should be fitted to the face without air-leaks.
- In general clinical setting (other than aerosol-producing procedures), a fluid-resistant mask is required. While fabric masks are widely available, this should not be used in any clinical setting. Figure 8 shows how to perform a particulate respirator seal check (72).

### Donning of PPE

**Figure 9:**
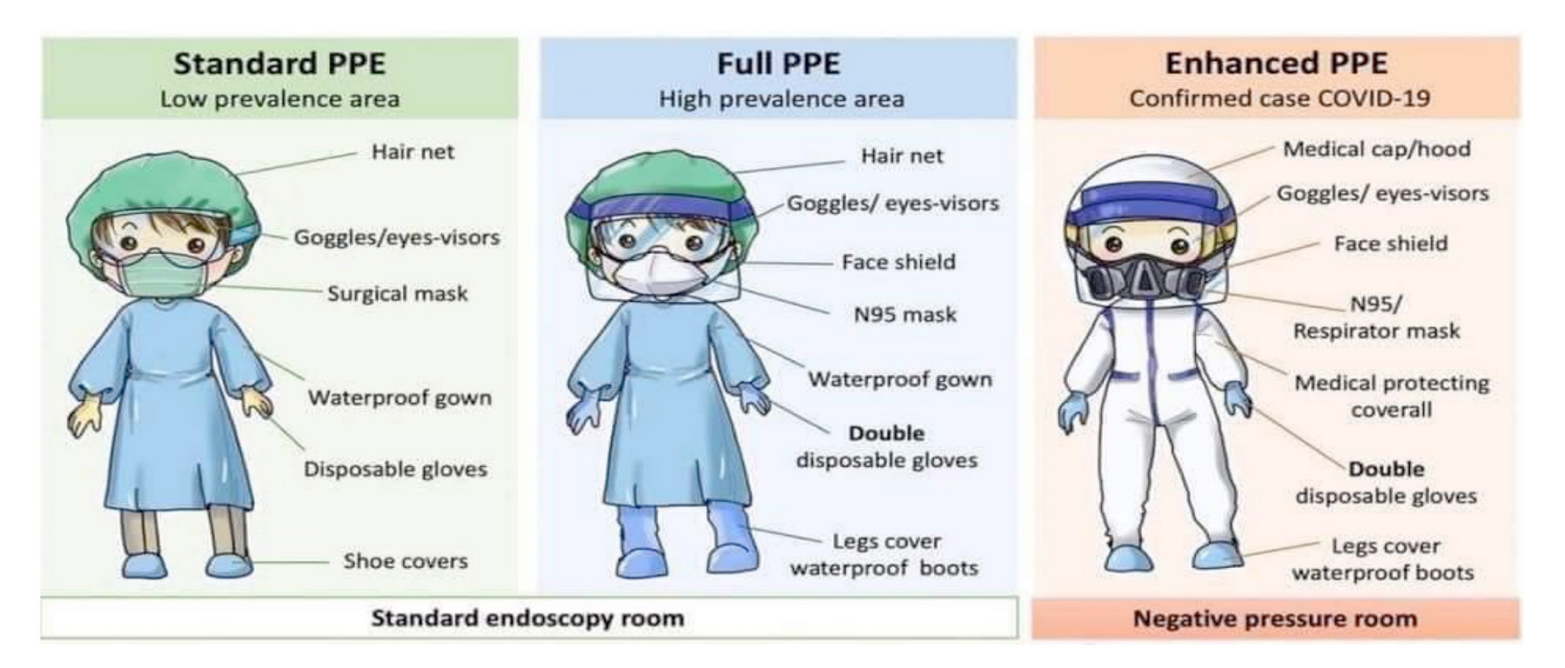
Levels of PPE

**Figure 10:**
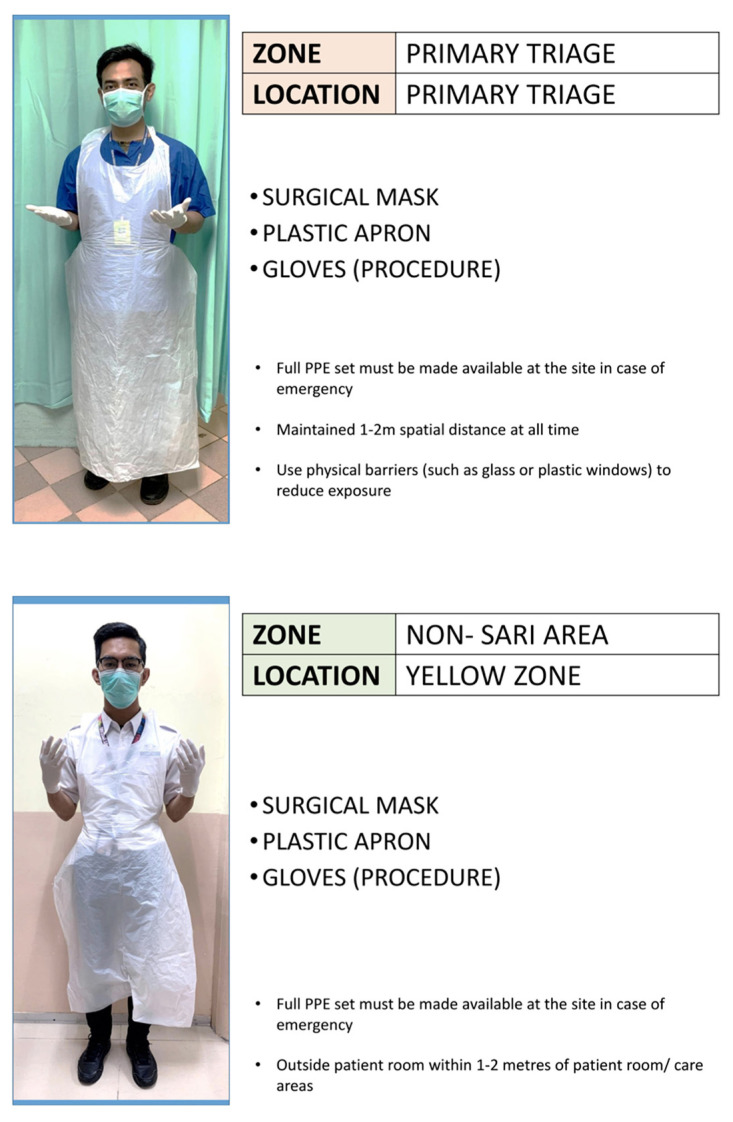
Recommended PPE to be used at the primary triage and non-SARI area

**Figure 11:**
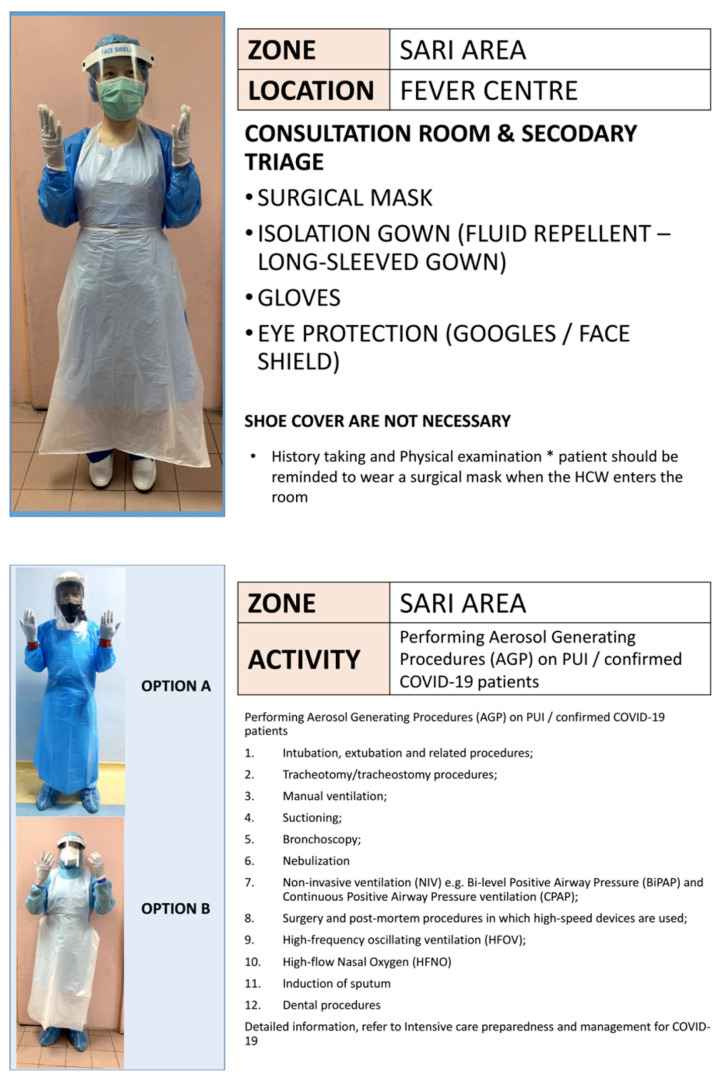
Recommended PPE to be used at the SARI area based on the location and activity

- Essential to sanitise hand with alcohol gel before, in between each step and after donning of PPE
- PPE must be put on in an order that ensures adequate placement of PPE equipment and prevents self-contamination and self-inoculation while using PPE and when taking off PPE (69, 71). Figure 9 shows levels of PPE. Figure 10 shows the recommended PPE to be used at the primary triage and non-SARI area, while Figure 11 shows the recommended PPE to be used at the SARI area based on the location and activity
- Collect all equipment needed and ensure donning done in the designated area
- Put on fluid-resistant boots and covers
- Put on inner disposable gloves
- Put on a disposable protective medical gown
- Cuffs of the gown goes over the inner gloves
- Put on medical protective masks
- For high risk procedures, use a surgical mask with PAPR. If not available, wear an N95 mask or equivalent/higher level with a full-face visor
- Perform respirator seal check for N95 mask
- Put on outer disposable gloves
- Ensure cuffs of gown is covered by outer gloves

### Doffing of PPE

- Essential to sanitise hand with alcohol gel before, in between each step and after doffing of PPE
- PPE has to be taken off in a manner that prevents self-contamination and self-inoculation with contaminated PPE (69, 73)
- Ensure doffing is done in the designated area
- Remove boots with covers if wearing them
- Remove covers of boots with boots together by touching only the inner part of the boots
- Avoid touching the outer part of the covers and boots
- Remove disposable protective medical gown with outer gloves
- Remove gown by rolling manoeuvre in an inside-out manner
- Avoid touching the outer and anterior part of the gown.
- Remove face or eye protector (i.e. visors, goggles or PAPR)
- With inner gloves, after sanitised with alcohol gel, pull the strap from behind and remove the protector away from face
- Do not touch the front part
- Remove protective masks
- If in the OT, remove the mask and inner gloves in the designated area
- If in the ward, keep the mask on until outside the patient’s room/bay/ward
- With the inner gloves, after sanitised with alcohol gel, pull the straps from behind and remove mask away from the face
- If wearing PAPR, remove PAPR by touching only the inner part and pull away from the face. Avoid touching the outer part
- Remove inner gloves. Sanitise hands with alcohol gel or wash with soap and water

### Precautions During Surgery and at Times of Shortage

In the OT, the ideal pressure system is a negative pressure room and if available, the recommended location is at the corner of the operating complex (57, 61). The theatre should have a separate entrance. For intubation, the surgeons and personnel that are not involved in intubation should wait outside of the OT until anaesthetic induction and intubation are completed (12, 15, 61). During surgery (except for endoscopic endonasal procedures), procedures involving high speed devices are considered as aerosol-producing procedures and are high risk. The number of personnel in an OT should be minimised (58) and full PPE should be applied. A N95 mask or masks that offer a higher level of protection should be used (28, 65, 66). Endoscopic endonasal procedures are not safe and should be avoided (28, 33). If the surgery cannot be postponed, consider a craniotomy or microscope-based transsphenoidal procedure.

During a shortage of PPE, the hospital or physicians are encouraged to minimise the use of PPE (16, 69). There are options available, such as considering telemedicine, where appropriate, to avoid direct contact with patients hence removing the necessity of PPE. The use of sterile gloves should be reserved only for procedures requiring sterility (61, 65, 66). All elective or non-urgent procedures, which usually require components of PPE should be delayed (65). Certain areas should be monitored with restricted access, such as areas where suspected or confirmed COVID-19 patients are being treated. Some activities that require to be done at proximity to the patient (e.g. at bedside) must be planned early and bundle them together to minimise the number of times entering the room. Visitors should not be allowed unless necessary, with restricted numbers and amount of time spent in the area. Visitors must have clear instructions and guidance when donning and doffing with strict hand hygiene (20, 61). The use of appropriate PPE should be prioritised (19, 61) and rationalised according to the risk of exposure and transmission dynamics of pathogens (air droplets, contact). Overuse of PPE will further impact on supply shortages.

### Type of Setting and Activity Requires Different Levels of PPE (Table 8)

The physician usually have to tailor the PPE usage based on the setting and activity being performed. Below are some recommendations:

- Extend the use of surgical gowns, masks and face protectors between patients with the same disease, who were confined in the same area without changing in between patients and whilst performing low risk procedures (14, 19, 61)
- Consider re-usable face protectors (i.e. visors or googles) (61, 65, 66)
- Essential to ensure appropriate cleaning and disinfection between uses. For examples, the gowns/protectors should be discarded if damaged, or should be removed and cleaned and disinfected immediately if visibly soiled
- When there is shortage of PPE, prioritise these for aerosol-generating procedures, or activities where sprays or splashes are anticipated (61, 70, 71)
- When using a non-fluid-resistant protective gown, ensure a plastic apron is used beneath (65)
- Consider using components of PPE beyond the manufacturer-designated shelf-life (69)
- Centralised management of PPE supply (61)
- Monitor end-to-end distribution of PPE
- Ensure appropriate distribution of PPE supply to limit wastage and imbalance in supply

### Role of Ultraviolet Light for Sanitisation and Hand Hygiene

The ultraviolet (UV) spectrum is best known for UVA, UVB and UVC (germicidal radiation). The spectral ranges for UVA, UVB and UVC are 315 nm–400 nm, 280 nm–315 nm and 100 nm–280 nm, respectively. UVC is the one with the strongest antimicrobial/antiviral properties (74, 75). With the rising healthcare awareness, some industry has demonstrated the effectiveness of radiation disinfection, especially UV light disinfection system on surface contamination, such as floors and equipment after the manual chemical disinfection process is completed (76). UV light disinfection is an implementation of ‘no-touch’ technology, is chemical free, does not require changes in the room’s ventilation and will not leave a residue after treatment. In healthcare facilities such as ICU and OT, this may be an adjunct to disinfection process (77).

A laboratory study has shown that coronavirus could effectively be inactivated by UVC light (74, 75, 78). Nevertheless, the UV light device is not a substitute for handwashing, mask-wearing and distancing. Moreover, the International Commission On Non-Ionizing Radiation Protection (ICNIRP) does not recommend the usage of lamps in the home. This is due to lack of adequate instructions of installation, duration of disinfection and increasing cases of skin and eye burns (79). WHO has come out with a fact that UV lamps should not be used to disinfect hands or other skin areas. Furthermore, reiteration was made that ‘cleaning your hands with alcohol-based hand rub or washing your hands with soap and water are the most effective ways to remove the virus’ (80).

## Efficacy of Televideo Consultation in Clinical Cases

The Malaysian Medical Council has formed an advisory group to define and monitor Virtual Consultation (during the COVID-19 pandemic). This advisory is guided by the Medical Act 1971 (amended 2012) which regulates the registration and practice of medicine in Malaysia and the Malaysian Medical Council’s Code of Professional Conduct. A virtual consultation is a form of Telemedicine. Telemedicine (teleconsultation, video conferencing, teleworkers, televideo) is a medical service provided remotely via information and communication technology. When the consultation is conducted without physical contact and does not necessarily involve long distances, then it is known as remote consultation. The role of the Council is to regulate physicians, not technology. The Council reminds physicians that the use of technology does not alter the ethical, professional and legal requirements in the provision of care. The Malaysian Medical Council’s jurisdiction is within this country only and physicians must ensure appropriate liability protection is in place to provide indemnity for malpractice.

When the health care delivery is affected by any national epidemic or global pandemic, or any other movement restrictions imposed on the public by the government, the use of communication technology can improve the access to care. In this unprecedented time where the situation is seen to be very urgent, rapidly changing and where there is a fine balance between public safety and individual health, it is equally important for medical practitioners to have the virtues of accountability and truth telling. The Code of Professional Conduct clearly says a physical examination is ethically mandatory. A non-physical contact virtual consultation makes a physical examination incomplete other than the visual and auditory observation. However, if a physician under current circumstances conducting such telemedicine virtual consultation feels this is so in good faith, then appropriate treatment can be initiated based on such, without the need for a physical examination in person.

### Ethical Practice in Telemedicine

In providing medical care using telecommunications technologies, physicians are advised that they must possess adequate training and competency to manage patients through telemedicine. The ethical and legal requirements such obtaining valid informed consent from the patient should be taken, at the same time ensuring that the physician’s identity, place of practice and registration status are made known to the patient, and the identity of the patient is confirmed at each consultation. The identities of all other participants involved in the telemedicine are disclosed and approved by the patient, and documented in the patient record. Both the physician-site and the patient-site are using appropriate technology that complies with legal requirements regarding privacy and security and accreditation standards where required.

Considerations must be given to safety and maintaining a high standard of patient care. The physicians must consider whether the telemedicine medium affords adequate assessment of the presenting problem, and if it does not, an arrangement for a timely in-person assessment should be taken. The physician should be prepared to advise remote patients about how and where to arrange for necessary care when follow-up is indicated. With the limitation of telemedicine, the physicians should exercise caution when providing prescriptions or other treatment recommendations to patients whom they have not personally examined. When carrying out a diagnostic evaluation or prescribing medication, a physician conducting a remote interaction should: i) verify the patient’s identity; ii) confirm that the remote interaction is appropriate to the patient’s situation and medical needs; iii) write any prescriptions in keeping with best practice guidelines and formulary restrictions (and in keeping with ethics guidance on prudent stewardship); and iv) document the clinical evaluation and prescription, as well as any instructions given to the patient. A medical record of the consultation, in accordance with professional and legal requirements, are kept and available to other health care professionals for the provision of ongoing patient care. This is especially important when there is a follow-up and referral to other specialities. Hence, the physician must ensure adherence to the same obligations for patient follow up in telemedicine as is expected with in-person consultation.

Many centres have implemented the telemedicine in neurosurgical consultation, specifically in patients with confirmed COVID-19, or recovered patients (PCR negative and beyond 14 days incubation period) that may need comprehensive clinical assessment to be performed. Throughout all levels, neurosurgeons are encouraged to convert meetings (with staff, colleagues and patients) to teleconsultation and/or video conferencing (6). Teleconsultation minimises face-to-face clinic visits for all doctors and patients, including neurosurgeons and their patients (3). Staff and patients over the age of 65 are encouraged to avoid coming to the hospital and clinic. The conversion of many clinic visits as medically appropriate to this new modality allow patients to stay safe at home and allows clinic nurses and staff to help care for COVID-19 patients.

Another speciality that is using telemedicine is neurology, mainly for the assessment of patients with a suspected stroke. The telemedicine should enable the physician to discuss the case with the assessing clinician, talk to the patient and/or family/carers directly and review radiological investigations (50). Hence, it is important that a high-quality video link is maintained to enable the remote physician to observe the clinical examination. The physician providing care (at both ends of the system) should be appropriately trained in the hyperacute assessment of people with suspected acute stroke, in the delivery of thrombolysis and the use of this approach and technology. The impact on the quality of care, efficacy of telemedicine and decision-making using telemedicine should be regularly audited (81) in keeping with physician’s fiduciary obligations to patients across that continuum (82).

Timely updates from trusted sources about the relative risk of contracting the novel disease *versus* a more common one are critical (83, 84). Strategic social media use (e.g. hashtags) may be an effective way for agencies to communicate accurate information to the public during times of crisis. Residents may be advised to connect with and follow local health agencies and service providers for the most geographically relevant information. Researchers may use publicly available ‘big data’ (e.g. localised tweets) to gauge the risk of communication efforts of local agencies.

### Telemedicine in Other Services/Department (84–86)

In the emergency department, there is a surge control for ‘forward triage’, meaning utilisation of sorting the patients before they arrive in the emergency department. This allows patients to be efficiently screened, is both patient-centred and conducive to self-quarantine, and it protects patients, clinicians and the community from exposure. The physicians and patients are still able to communicate 24/7, either by using smartphones or webcam-enabled computers. The respiratory symptoms (which may be early signs of COVID-19) are among the conditions most commonly evaluated with this approach. Telemedicine consultations for Oncologic patients may not be suitable and individual clinicians must be able to make an appropriate judgment. However, patients will greatly benefit from such virtual clinic consultations over a cancellation.

## Neurosurgical Infection

### COVID-19 (87, 88)

The presence of virus within CSF fluid and during autopsy can be tested via electron microscopy, immunohistochemistry and real-time reverse transcriptional. There are 36.4% of patients had neurologic manifestations due to neurotropic potential in the COVID-19 virus found in one study. During an early or later phase of the infection, the dissemination of COVID-19 in the systemic circulation or across the cribriform plate of the ethmoid bone takes place. The ability to cross the blood brain barrier into the cerebral circulation is due to the properties of the COVID-19 virus spike protein with angiotensin-converting enzyme 2 (ACE2) receptors expressed in the capillary endothelium. The receptor has been detected over glial cells and neurons. COVID-19 virus exploits the ACE2 receptors to gain entry inside the cells (89) causing neuronal death in mice by invading the brain via the nose close to the olfactory epithelium. In an uncomplicated early stage, findings like an altered sense of smell or hyposmia can be found. Once the virus caused respiratory manifestation, there will be neurological involvement with loss of involuntary control over breathing (90).

### Tuberculosis (91)

The physician should maintain continuity of essential services for people affected with Tuberculosis (TB) during the COVID-19 pandemic. It is anticipated that ill patients with both TB and COVID-19 may have poorer treatment outcomes, especially if TB treatment is interrupted. Therefore, accurate diagnostic tests are essential for both TB and COVID-19. Simultaneous testing of the same patient for both TB and COVID-19 would generally be indicated for three main reasons (subject to the specific setting in the country): i) clinical features that are common to both diseases; or ii) simultaneous exposure to both diseases; or iii) presence of a risk factor for poor outcomes to either disease. In tuberculous meningitis with communicating hydrocephalus, the recommendation is to treat with furosemide with or without acetazolamide. Some institutions favour daily lumbar punctures with ICP monitoring through manometry (92). In tuberculous meningitis with noncommunicating hydrocephalus, this will involve invasive neurosurgical procedures such as an external ventricular drain (EVD), ventriculoperitoneal shunting or endoscopic third ventriculostomy (ETV) (92).

### Acute Necrotising Encephalopathy

Acute necrotising encephalopathy is a condition that can be triggered by viral infections like influenza and herpes. A case report of a woman who tested positive for COVID-19 developed acute necrotising encephalopathy (93). The patient presented altered mental status and a noncontrast head CT images demonstrated symmetric hypoattenuation within the bilateral medial thalami with a normal CTA and CT venogram. Brain MRI demonstrated a haemorrhagic rim enhancing lesions within the bilateral thalami, medial temporal lobes, and subinsular regions. The patient was started on intravenous immunoglobulin.

### Viral Encephalitis

This is the first reported case of meningitis associated with SARS-CoV-2 who presented with convulsion followed by unconsciousness (94). The patient had transient generalised seizures that lasted about a minute. The specific SARS-CoV-2 RNA was not detected in the nasopharyngeal swab but was detected in a CSF. Brain MRI showed hyperintensity along the wall of right lateral ventricle and hyperintense signal changes in the right mesial temporal lobe and hippocampus, suggesting the possibility of SARS-CoV-2 meningitis.

## Paediatric Neurosurgical Emergencies

Paediatric neurosurgery accounts for up to 15% of neurosurgical admissions. In this current crisis, COVID-19 infection among children remains limited in numbers. However, paediatric patients are at risk of exposure from potential family members and caretakers that may harbour the virus and remain asymptomatic. The main consideration is therefore to be vigilant and screen caretakers and family of patients for risk of exposure based on current and updated guidelines provided by the MOH (1). The priority as for all cases in the pandemic crisis remains the protection of healthcare personnel and other non-infected patients as well as the working environment at healthcare facilities.

The guidelines provided by MOH (1) applies equally for paediatric neurosurgery cases; principally in deciding for the need of surgical care and intensive management in times of reduced availability of resources. This guiding principle forms the core basis of decision making for paediatric neurosurgery in Malaysia as the availability of specialised paediatric intensive care units and specialised instruments are typically present in major hospitals with dedicated neurosurgical units and facilities. With the ongoing pandemic crisis, these subspecialised resources are crucial for the treatment of patients afflicted by COVID-19.

### Clinic/Outpatient Services (95)

All non-essential outpatient cases should be postponed. Some new cases with symptoms and signs of raised ICP or neurological compromise should be reviewed urgently by a neurosurgeon. Whereas, new cases with no symptoms or signs of raised ICP or neurological compromise should be reviewed via teleconsultation. Any pending surgeries should be reviewed with team and MDT to prioritise cases based on the urgency of surgical intervention, taking into account symptoms and signs, radiological evidence of mass effect or vital structure compression, expected radiological progression over time and expected histology along with the availability of vital specialised resources of intensive care, equipment requirements and OT availability. Certain logistic factors, such as patient travel and family economic factor that requires one or both parents to be away from work should also be taken into account in the decision making process to ensure compliance of patient and caretakers to the management plan.

### Paediatric Neurotrauma (38)

All cases must be screened for risk of COVID-19 as per hospital protocols. The management of paediatric neurotrauma is based on the Brain Trauma Foundation (BTF) guidelines. All cases undergoing emergency surgery must be performed under full PPE as per hospital/MOH protocols (1, 62). The initial referral should be reviewed by the neurosurgical team or consulted via teleconferencing with the neurosurgical team on admission. The modified paediatric GCS score system (Table 9) must be applied and made available to referring or primary management team where applicable (96). Cases of neurotrauma with surgical lesions must be transferred to a hospital with a neurosurgical facility and or services for management. Frequent repeats of CT scan should be avoided where feasible and should be factored into the decision making process for applicable cases.

There are certain cases of neurotrauma that has positive CT findings but with a non-surgical lesion (97). If the treatment requires an ICP monitoring, then the patient should be transferred to a centre with neurosurgical services or facilities. If the treatment does not require an ICP monitoring but there is a potential risk of progression and deterioration, then the patient should also be transferred over. However, for patients who do not require intensive care with low risk of deterioration or progression, it is advisable to transfer to centres with available neurosurgical services. If it is not feasible, then the patient should be managed by attending paediatrician/surgeon at primary referral centres with regular consultations to the neurosurgical team. Cases with no positive CT brain findings (97) may be managed at primary referral centres if deemed suitable with a consultation to the neurosurgery team.

In a non-traumatic paediatric neurosurgery case, the approach is the same; all cases must be screened for risk of COVID-19 as per hospital protocols. Suitable use of PPE must be adhered to for all cases undergoing surgery. The surgeon should: i) consider the option of fastest and simples access route to the lesion; ii) consider biopsy if deemed appropriate over surgical resection; iii) consider the use of adjuncts such as image guidance and/or ultrasound guidance to facilitate quick and effective surgery if available; and iv) consider potential prolonged PICU support in case selection to avoid prolonged ICU/hospital stay.

The priority of surgery is divided into: i) urgent; ii) emergency/early surgical intervention; and iii) delayed surgery. Urgent surgical intervention includes cases with symptoms and signs of raised ICP with a mass lesion (5, 37, 98), such as:

- Acute hydrocephalus — consider ventriculoperitoneal (VP) shunt at first surgery if deemed appropriate to avoid the need for repeat surgery. However, if ETV is planned, then the recommendation is to use the ETV success score (if < 70 to consider for VP shunt) and consider insertion of rescue EVD in cases of potential failure of ETV
- Lesions causing neurological compromise from compression of vital structures such as vision and motor function
- Open craniospinal dysraphism
- Spinal instability from any cause with progressive neurological deficits
- Acute CNS infections such as empyema and abscess
- For surgery with the aim to repair an anterior cranial fossa CSF leak, a full PPE precaution is mandatory for all personnel

The emergency/early surgical intervention should be performed within 1–2 weeks. These include:

- All symptomatic posterior fossa tumours with or without hydrocephalus; treatment of hydrocephalus should be considered independent of the tumour of deemed appropriate by attending neurosurgical team
- Intraventricular tumours with hydrocephalus
- All symptomatic supratentorial brain tumours with mass effect
- All malignant brain tumours – consider biopsy for cases where adjuvant therapy deemed appropriate
- All symptomatic spinal cord tumours
- For Pineal region tumours, consider ETV and biopsy as first line surgical approach

Delayed surgery is considered within a 3 months period. These include:

- Low grade tumours with no mass effect; asymptomatic or remain stable in symptoms and radiologically appearance
- Closed craniospinal dysraphism
- Surgery for epilepsy if seizure control adequate with medications
- Functional neurosurgery including DBS, Intrathecal baclofen pump and vagal nerve stimulator
- Craniofacial surgery
- Surgery involving paranasal sinuses

## Figures and Tables

**Table 1:** Tiers for neurosurgery cases

Tier	Action	Definition	Locations	Examples
Tier 1a	Postpone surgery/procedure	– Low acuity surgery/healthy patient – Outpatient surgery – Not life threatening illness	HOPD ASC Hospital with low/no COVID-19 census	– Carpal tunnel release – Colonoscopy – Cataracts
Tier 1b	Postpone surgery/procedure	Low acuity surgery/unhealthy patient	HOPD ASC Hospital with low/no COVID-19 census	– Endoscopies
Tier 2a	Consider Postponing surgery/procedure	– Intermediate acuity surgery/healthy patient – Not life threatening but potential for future morbidity and mortality. Requires in-hospital stay	HOPD ASC Hospital with low/no COVID-19 census	– Low risk cancer – Non urgent – Ortho: Including hip and knee replacement – Non urgent spine: elective spine surgery – Stable ureteric colic – Elective angioplasty
Tier 2b	Postpone surgery/procedure if possible	Intermediate acuity surgery/unhealthy patient	HOPD ASC Hospital with low/no COVID-19 census	
Tier 3a	Do not postpone	High acuity surgery/healthy patient	Hospital	– Most cancers – Neurosurgery/emergency spine surgery – Highly symptomatic patients
Tier 3b	Do not postpone	High acuity surgery/healthy patient	Hospital	Transplants Trauma Cardiac w/symptoms limb threatening vascular surgery

Notes: HOPD = Hospital Outpatient Department; ASC = Ambulatory Surgery Centre

**Table 2:** Distribution of neurosurgery operative cases

Surge level	Emergent and urgent cases	Elective and procedural cases	Transfers
**Green:** 1–9 acommunity cases, or < 6 COVID+ inpatients and no staffing shortages	– Ensure OTs and surgeons available to rapidly operate and discharge ED cases (appy/chole/hip fx) – Maintain capacity for urgent cases	– Proceed – Review and schedule all cases 7 days in advance for quick cancellation should surge level increase to yellow	Per service protocol
**Yellow:** 10–99 community cases or 7–16 COVID+ inpatients or < 20% staffing shortages	– Ensure OTs and surgeons available to rapidly operate and discharge ED cases (appy/chole/hip fx) – Maintain capacity for urgent cases	– Cap OT schedule for next 3 weeks – 25% reduction in procedural cases requiring overnight stay (ERCP/cath/IR) – Ramp up come and go procedures at outpatient facility – Reschedule OT cases so no more than 25 post-op admits over next 7 days	Time sensitive protocol
**Red:** > 100 community cases, or > 17 COVID+ inpatients or > 21% staffing shortages	– Ensure OTs and surgeons available to rapidly operate and discharge ED cases (appy/chole/hip fx) – Maintain capacity for urgent cases	– Cap OT schedule for next 3 weeks – 50% reduction in procedural cases requiring overnight stay (ERCP/cath/IR) – No use of PACU for recovery – Cancel come and go procedures – Reschedule OT cases so no more than 12 post-op admits over next 7 days	Closed to all transfers
**Black:** Significant assistance needed from outside institution	Emergent cases only	Cancel all scheduled cases	Closed to all transfers

Note:

a Community cases refer to active cases in the local region.

OT = operating theatre; ED = emergency department; appy = appendectomy; chole = cholecystectomy; hip fx = hip fracture; ERCP = endoscopic retrograde cholangiopancreatography; IR = interventional radiology; cath = cardiac

**Table 3:** Group setting (surgical team)

Site 1: Main hospital			Site 2: Major hospital			Site 3: Trauma hospital			Site 4: Pediatrics hospital			
Team 1	Team 2	Backup	Team 1	Team 2	Backup	Team 1	Team 2	Backup	Team 1	Team 2	Backup	Additional back-up coverage
Surg	Surg	Surg	Surg	Surg	Surg	Surg	Surg	Surg	Surg	Surg	Surg	Fellow (spine)
MO	MO	MO	MO	MO	MO	MO	MO	MO	MO	MO	MO	Fellow (peripheral nerve)
PGY-2	PGY-2	PGY-2	PGY-4	PGY-2	PGY-3	PGY-2	PGY-4	PGY-3	PGY-4	PGY-4	PGY-4	Fellow (vascular)
PGY-3	PGY-3	PGY-3										Fellow (oncology)
PGY-4	PGY-4	PGY-4										

Notes: Peds = pediatric; Surg = surgeon; Mo = medical officer; PGY = post-graduate year (resident level)

Source: Burke JF et al. (6)

**Table 4:** Summary of head and neck examination and procedure recommendations

Risk and definition	Patient wears	Clinician/staff wear
Non procedure encounters in non-immune-compromised patients		
High risk to clinician: any examination in:	Surgical mask	Single-use N95 mask
Patients with active SARS-CoV-2 infection		Goggles or face shield
Patients with influenza like sympotoms		Gown
Patients under evaluation for SARS-CoV-2 infection		Gloves
Moderates risk to clinician: examination of ear, nose, mouth, or throat in asymptomatic patients	Nothing^a^	Surgical mask with face shield to allow for reuse of mask
		Gloves
Low risk to clinician: other examination in asymptomatic patients	Nothing^a^	Mask optional
		Gloves
Non-aerosol-generating interventional procedures		
Soft tissue surgery exposes blood, which can have a viral count, but unless the blood is aerosolised by the use of energy devices, it would be expected to be lower risk. Suctioning away smoke and aerosolised tissue is recommended. To our knowledge, the infectiousness of aerosolised blood with SARS-CoV-2 is not yet known		
High risk to clinician: consider delaying or discussing in:	Surgical mask	Single-use N95 mask
Patients with active SARS-CoV-2 infection		Goggles or face shield
Patients with influenza like symptoms		Gown
Patients under evaluation for SARS-CoV-2 infection		Gloves
Low risk to clinician: patients who are asymptomatic or SARS-CoV-2 negative in last 48 h	Nothing^a^	Surgical mask
		Goggles or face shield
		Gown
		Gloves

Notes:

a If the patient is immune compromised (receiving active chemotherapy, radiotherapy, or immunotherapy, < 1 year after solid organ transplant; receiving chronic immunosuppression therapy; pregnant), both the patient and clinician should wear surgical mask unless the patient is high risk. Clinicians and staff should wear a face shield over and surgical mask to allow reuse of mask.

Source: Givi B et al. (28)

**Table 5:** Case series of open tracheostomies performed during the severe acute respiratory syndrome (SARS) outbreak

Characteristic	Case series		
	Chee et al. (30)	Tien et al. (31)	Wei et al. (32)
Institution	Tan Tock Seng Hospital, Singapore	Sunnybrook and Women’s College Health Sciences Centre, Toronto, Canada	Queen Mary Hospital, Hong Kong SAR, China
No. of tracheostomies performed	15	3	3
Barrier precautions during surgery	aStandard PPE, shoe covers, and powered air-purifying respirator system	aStandard PPE and Stryker T4 Protection System	aStandard PPE, shoe covers, and additional plastic face shield worn outside goggles
Setting of surgery	Negative-pressure room in ICU	Negative-pressure room in ICU	Negative-pressure room in ICU or operating room
Intraoperative steps to reduce aerosolisation	Complete paralysis of the patient, mechanical ventilation stopped before tracheostomies; limited suction used during the procedure, no specific avoidance of diathermy other than during btracheostomy	Complete paralysis of the patient, mechanical ventilation stopped before tracheostomy, no suction used after trachea was entered, diathermy avoided when possible	Complete paralysis of the patient, mechanical ventilation stopped before tracheostomy, no suction used throughout the procedure, diathermy avoided as much as possible
Surgical team members	Single dedicated team performing all tracheostomies: experienced surgeon, experienced anesthesiologist, 1 scrub nurse and 1 bassistant	Senior attending trauma surgeon and most senior surgical staff member available, attending ICU anesthetist, and no circulating nurse or scrub nurse	Single surgeon, 1 intensive care specialist, and 1 standby medical or nursing staff member

Notes:

a Standard PPE consists of an N95 mask, surgical cap, goggles, surgical gown, and gloves.

b Personal experience from Mark Li-Chung Khoo, MBBS (February 23, 2020), surgical lead for tracheostomies at Tan Tock Seng Hospital during the SARS outbreak.

Source: Tay et al. (29).

**Table 6:** Proposed recommendations for management of brain trauma in pandemic crisis

**Hospitals with neurosurgical services with dedicated neurosurgical facilities available**	Brain trauma with surgical lesion and mass effect requiring urgent surgical intervention(decompression) with or without intensive care monitoring	Level II
	Brain trauma with non-surgical lesion, requiring or benefit from ICP monitoring and intensive care management	Level II
	Brain trauma requiring multidisciplinary management	Level II
	Brain trauma with no surgical lesion and not requiring intensive care management but risk of deterioration is deemed high risk	Level II/III

**Hospitals with neurosurgical services without dedicated neurosurgical facilities available**	Brain trauma with surgical lesion and mass effect requiring urgent surgical intervention (decompression) with or without intensive care monitoring	Level II
	Brain trauma with non-surgical lesion managed with option of CPP guided medical therapy with serial CT scan at 6–12 h intervals in intensive care setting	Level III
	Brain trauma requiring surgical intervention but with limited resources available:	Level III
	Surgical intervention in neurosurgical facilities with subsequent transfer back to primary referring hospitals for continuity of intensive care management	
	Brain trauma with non-surgical lesion with risk of progression or deterioration deemed as high risk	

**Hospitals with no available neurosurgical services or facilities general surgical services available**	Brain trauma requiring surgical intervention but with limited resources available:	Level III
	Deployment of neurosurgical team to primary referral hospital where feasible to facilitate timely intervention	
	Surgical intervention in neurosurgical facilities with subsequent transfer back to primary referring hospitals for continuity of intensive care management	
	Surgical intervention and management undertaken by trained and privileged general surgery team for selected cases	Local/Regional practice
	Brain trauma with non-surgical lesion with low risk of progression or deterioration	

Source: Malaysia Health Technology Assessment Section (MaHTAS) (39)

**Table 7:** Categorising elective neurosurgical cases

Categorising Elective Neurosurgical Cases	
**Category 1**	Brain tumours Intra axial space occupying lesion with minimal and/slow progressing symptoms Extra axial symptomatic benign tumours (schwannoma, meningioma, craniopharyngioma, cysts) without cerebral oedema Pituitary tumour with stable visual deficit and/or symptomatic endocrine dysfunction Parasellar or clival lesion with deteriorating neurological deficit and adjacent oedema Neurovascular disorders Asymptomatic aneurysm/incidental finding with no history of bleed or acute neurological deficit CSF diversion procedures Previously diagnosed hydrocephalus with evidence of raised intracranial pressure Newly diagnosed hydrocephalus on CT or MRI Patient with complications or suspected complications of an in-situ shunt Idiopathic intracranial hypertension – in patients with persistent symptoms or visual deterioration despite medical therapy including repeat lumbar punctures Non-acute traumatic brain injury Non-acute skull fractures Non-acute traumatic brain injury Spine/Neck/Back pain Irreversible deficit if not seen within 1–4 weeks Significant spinal nerve root compression or spinal cord compression with slower evolving neurological signs/symptoms Severe pain with significant functional impairment Moderate to severe sciatica with recent reflex and muscle power deficit eg. foot weakness Moderate to severe neck pain and arm pain with recent reflex and muscle power deficit Peripheral Nerve Compression Carpal tunnel syndrome or severe ulnar entrapment neuropathy with weakness/muscle wasting and electrophysiological confirmation of diagnosis Peripheral nerve compression with neurological deficit or severe pain syndrome
**Category 2**	Brain tumours Functioning or non-functioning pituitary adenoma, pituitary tumours with slowly progressing visual deficit Parasellar tumour, clival lesion with slowly progressing neurological deficit Incidental lesional findings on imaging cyst, Chiari malformation, venous angioma Spine/Neck/Back pain Less severe and more long standing lain with significant functional impairment Acute cervical and lumbar disc prolapse with stable neurological signs/symptoms Severe degenerative spinal disorders with limitation of activity of daily living Acute cervical or lumbar disc prolapse with moderate to severe limb pain but minimal neurological deficit Documented severe lumbar canal stenosis with significant neurogenic claudication/limitation of walking distance Documented severe cervical canal stenosis with significant neurology over the upper limbs with limitation of hand functions Acute pars defect in young adult Anterolisthesis/spondylolisthesis with lower limb neurology and/or instability on flexion/extension X-rays
**Category 3**	Congenital Craniostenosis with or without slow neurological deficit Craniofacial disorder with or without slow neurological deficit Encephalocoele/meningocoele with or without slow neurological deficit Functional/Quality of life Spine/Neck/Back pain Mechanical lower back pain without lower limb pain Stable mild neurological symptoms/signs which is unlikely to progress if l left untreated or in whom a good surgical outcome is uncertain Pain that is manageable or reasonably controlled with analgesia Chronic low back pain/neck pain (without radiculopathy) Most cases of chronic cervical and lumbar disc prolapse and degenerative spinal disorders without or with stable mild deficit Long standing spondylolisthesis with stable neurology Peripheral nerve compression – Ulnar nerve entrapment neuropathy when not responding to > 6 months of maximal medical management Trigerminal neuralgia/hemifacial spasm – When not responding to > 6 months of maximal medical management Seizure – When not responding to > 6 months of maximal medical management Movement disorder – When not responding to > 6 months of maximal medical management Psychiatry disorder –When not responding to > 6 months of maximal medical/psychiatric management Corrective – Cranioplasty for trauma and non-trauma skull defects Consideration should be given to the available resources, facilities, equipment, consumable and real time logistic capability and feasibility. Designated COVID-19 hospitals may not be able to support all elective cases, in particular those that require post-operative intensive care or significant use of blood and blood products Surgeons, in consultation with anaesthetist, nursing colleagues as well as patients (or legally accepted next of kin), should weigh the risks of proceeding (exposure, lack of resources) against those of deferment, (progression of disease, worse patients outcomes) including the expectation of delay of 2–3 months or more or until the COVID-19 is less prevalent Elective surgery acuity scales (ESAS) St. Louis University, adapted from ACS (tier classifications) Benefit of intra-operative neurophysiological monitoring, which surgery should be monitored? More personnel within the operation theatre if its used. Minimally invasive procedures (tubular system, scope assisted, percutaneous) versus open technique Choice of anaesthesia and awake versus asleep procedures Special precautions for surgery in which nasal and oral excess is needed, extra care needs to be taken while performing such surgery as it involves patient’s upper respiratory tract (which is primarily the highway for COVID-19) infection. Strongly recommend to delay any non-urgent such surgery to reduce the risk of exposure

Source: Ministry of Health Malaysia (56)

**Table 8:** Levels of PPE required in different clinical settings

Application	Personal protection equipment required
– In outpatient setting	Level I protection – Disposable surgical cap – Disposable surgical mask – Disposable non-sterile latex gloves
– Isolated ward, HDU or ICU area – Non-respiratory examination of suspected or confirmed patients – Cleaning of surgical instruments used for suspected or confirmed patients	Level II protection – Disposable surgical cap – N95 mask, equivalent or mask which offer higher level of protection. – Disposable protective medical gown – Disposable latex gloves, double gloving – Face protector (visor or googles)
– Performing surgical procedures on suspected or confirmed patients during which respiratory secretions, bodily fluid or blood spray or splash is expected to occur or high-speed devices are used.	Level III protection – Disposable surgical cap – Powered air-purifying respirator (N95 mask, equivalent or mask which offer higher level of protection with full face visor if PAPR is not available) – Disposable medical protective gown – Disposable sterile latex gloves, double gloving – Protective rubber boots with disposable boots covers

Source: Ti LK et al. (15)

**Table 9:** The modified paediatric GCS score

	Score	Standard GCS	Paediatric GCS
Eye opening	4	Spontaneous	Spontaneous
	3	To voice	To voice
	2	To pain	To pain
	1	None	None
Verbal response	5	Oriented	Coos/babbles
	4	Confused	Irritable/cries
	3	Inappropriate words	Cries to pain
	2	Incomprehensible sounds	Moans
	1	None	None
Motor response	6	Follows commands	Spontaneous movement
	5	Localises pain	Withdraws to touch
	4	Withdraws to pain	Withdraws to pain
	3	Abnormal flexure posturing	Abnormal flexure posturing
	2	Abnormal extension posturing	Abnormal extension posturing
	1	None	None

Source: Borgialli et al. (96)
